# The effects of diabetes self-management programs on clinical and patient reported outcomes in older adults: a systematic review and meta-analysis

**DOI:** 10.3389/fcdhc.2024.1348104

**Published:** 2024-06-17

**Authors:** Paige Alliston, Milos Jovkovic, Saira Khalid, Donna Fitzpatrick-Lewis, Muhammad Usman Ali, Diana Sherifali

**Affiliations:** ^1^ Faculty of Health Sciences, School of Nursing, McMaster University, Hamilton, ON, Canada; ^2^ Department of Clinical Epidemiology & Biostatistics, Faculty of Health Sciences, McMaster University, Hamilton, ON, Canada; ^3^ Population Health Research Institute, Hamilton Health Sciences, McMaster University, Hamilton, ON, Canada

**Keywords:** self-management, diabetes, older adults, systematic review, meta-analysis

## Abstract

**Objectives:**

With diabetes self-management continuing to become more complex for older adults, self-management programs have been shown to support this population in meeting their multifaceted medical needs. Building on our previous systematic review and meta-analysis, we aimed to update the literature on the effectiveness of diabetes self-management programs and investigate the impact of specific self-management interventions on clinical and patient-reported outcomes.

**Methods:**

We updated our literature search in the following databases: Medline, EMBASE, PsychINFO, CINAHL and Cochrane Database of Randomized Controlled Trials from November 2013 to July 2023 for studies that may fit our inclusion criteria. Two independent reviewers screened and extracted data from the included group of studies.

**Results:**

A total of 17 studies with 21 comparison arms met the inclusion criteria, totalling 5976 older adults (3510 individuals randomized to self-management programming and 2466 to usual care). The pooled effectiveness of diabetes self-management programs in older adults on glycemic control (hemoglobin A1C) was a reduction of -0.32 (95% CI -0.44, -0.19). Specifically, the most effective approach on glycemic control (A1C) was the use of feedback (-0.52%; 95% CI -0.68, -0.36). Overall, self-management programs improved behaviour change outcomes, with feedback interventions being most effective (standardized mean difference [SMD] 0.91; 95% CI 0.39, 1.43). The effect of self-management programs on body mass index, weight and lipids were statistically and clinically significant.

**Conclusions:**

The evidence for diabetes self-management programs for older adults demonstrates a small but clinically meaningful reduction in A1C, improvement in patient-reported outcomes (behaviour, self-efficacy, knowledge), and other clinical outcomes (BMI, weight and lipids). The specific strategy used in diabetes self-management programs for older adults should be considered to achieve optimal results on outcomes.

## Introduction

It is estimated that 1 in every 5 people between the ages of 65-99 years old, or 136 million people, live with diabetes worldwide (1). This number is projected to rise to 276 million by 2045 (1). As society ages, the healthcare burdens associated with diabetes in older adults will grow significantly (1, 2). This is in part due to this population managing comorbidities in addition to diabetes, including frailty, cognitive impairment, functional disability, and vascular complications, among others (2–5).

The vascular and non-vascular comorbidities that are often paired with diabetes in older adults present a unique set of challenges for this population to overcome when managing their condition (2). Older adults experiencing multimorbidity utilize health services more often (6, 7), experience poorer diabetes outcomes, and have a reduced quality of life due to their complex medical management (5). Social isolation and insufficient diabetes-related knowledge can lead to a decrease in performing self-management behaviours for this population, making them vulnerable to diabetes-related complications and premature mortality (4, 5, 8, 9).

Recent clinical practice guidelines emphasize the importance of individualized care plans to support diabetes management for older adults (10). As this population includes a spectrum of individuals ranging from clinically stable to functionally dependent or near the end of life, the approach to disease management must be personalized (10, 11). Social, functional, cognitive, medical, and psychological factors should be assessed to provide a framework for creating an individualized care plan (11). Overtreatment is a common occurrence for older adults, leading to an increased burden of managing diabetes (11) and may contribute to the negative feelings older adults experience towards self-care behaviours (8). Strategies including a simplified pharmacotherapy regimen and a more liberalized A1C target can be used to prevent hypoglycemia for older adults living with multiple comorbidities (10, 11). Self-management education and support for older adults is also an important component of diabetes care (10). Strategies to best address self-management and diabetes education for this population are warranted.

Since the completion of our previous systematic review (12), new literature has emerged on diabetes self-management education and support for older adults. In particular, the complexity of diabetes management for older adults elicits an investigation into the impact of these interventions on patient-reported outcomes such as quality of life, behaviour change, diabetes-related distress, diabetes knowledge, and self-efficacy. We aim to apply the same methodological approach as our previous review to examine more recent publications and determine the most effective diabetes self-management education or support strategies in older adults, as measured by glycemic control, metabolic outcomes, and patient-reported outcomes.

## Materials and methods

### Conceptual model

Aligning with the conceptual model applied to our previous review (12), the framework by Chodosh et al. (13) was used to describe self-management programs through five categories: tailoring; group setting; feedback; psychological emphasis; and medical care. Studies were characterized using the five elements based on the most dominant characteristic with the assumption that the outcomes are associated with that study characteristic.

### Search strategy

Relevant biomedical databases, including Medline, EMBASE, PsychINFO, CINAHL, and Cochrane Database of Randomized Controlled Trials were searched from November 2013 to July 2023 by applying the previous search strategy developed in consultation with a medical research librarian (12). Please refer to **Supplementary Table 1** for an overview of our search strategy. References from relevant guidelines, systematic reviews, and meta-analyses were also examined for relevant publications. The PRISMA checklist for systematic reviews was followed throughout the completion of our review (**Supplementary Table 2**).

### Study selection and quality appraisal

The eligibility criteria from our previous review were applied to assess all citations (12). Briefly, the inclusion criteria consisted of peer-reviewed literature, written in English, and (1) was a randomized, controlled trial; (2) included a population of older adults ≥ 65 years of age that live with Type 2 diabetes; and (3) report the differential effect of self-management support strategy or education on glycated hemoglobin A1C (HbA1c), metabolic outcomes (fasting blood glucose, body mass index [BMI], weight, lipids, blood pressure), or patient-reported outcomes (quality of life, behaviour change, diabetes-related distress, diabetes knowledge, self-efficacy). Publications were excluded if: (1) data was reported on subjects without diabetes, < 65 years of age; (2) the self-management education intervention included testing new or combination oral anti-diabetic agents; (3) no statement of informed consent being obtained was included.

Three research team members (PA, MJ, SK) reviewed citations independently at the title and abstract and full-text levels. Quality assessment and data extraction were completed by three independent team members (PA, DFL, MJ). Disagreements at the full text, data extraction, and quality assessment levels were resolved through discussion between team members. Data extraction was completed using standardized forms and included information on objectives, demographics, self-management interventions, and outcomes for each publication. Quality appraisal was completed using Cochrane’s risk of bias tool (14), and studies were ranked based on having a low, unclear, or high risk of bias.

### Data analysis

All data analyses were planned *a priori*. A meta-analysis was used to combine the results across studies by outcome using the published continuous data from included studies. Specifically, we used the change from baseline to immediate post-treatment or follow-up data (mean change score, standard deviation of mean change score) for both intervention and control groups. In the studies where a measure of variance was reported as confidence intervals, standard error or *p*-values, we used Cochrane-recommended methods to convert to standard deviation (15). We used a multi-level meta-analytical approach (where applicable) to account for statistical dependence i.e., dependency in effect sizes introduced either by comparison of multiple intervention arms within a study to a common control group or by multiple outcome measures or sub-outcome measures of a primary outcome of interest within a study (such as various measures of patient-reported outcomes) (16–18). The statistical heterogeneity I^2^ statistic was also estimated in the context of a multi-level meta-analytical approach i.e., within-cluster heterogeneity (multiple arms from the same study) and between-cluster heterogeneity (effect sizes across studies). Overall I^2^ for each summary effect size was estimated to represent the heterogeneity not attributable to sample error and is the sum of within-cluster and between-cluster heterogeneity (19).

The summary measures of effect were generated as mean difference (MD) and standardized mean difference (SMD). The units of measurement for clinical outcomes such as weight, lipid profile measures, and blood glucose were converted into the same standard units (mmol/L for lipid outcomes and glucose, and kg for weight) before input into metanalysis. The SMD was used as a summary statistic for PROMs because the studies in this systematic review often assessed the same outcome using different outcome measures or tools. Given this, it was necessary to standardize the results of the studies before they could be compared across studies or combined in a quantitative synthesis. The SMD is interpreted based on its magnitude according to Cohen *d* recommended thresholds (~0.2=small effect, ~0.5=medium effect, ~0.8=large effect) (20, 21).

Cochran’s Q (α=0.05) was employed to detect statistical heterogeneity and I² statistic to quantify the magnitude of statistical heterogeneity between studies where I² >50% represents moderate and I² >75% represents substantial heterogeneity across studies (22). To ascertain statistical stability and robustness of results, further meta-regression analysis was carried out based on pre-specified subgroups of interest where possible based on the evidence available i.e., the focus of intervention based on Chodosh framework (i.e., Tailoring, group, medical, feedback, and psychological emphasis) and intervention length of follow-up (< 6 months, 6 months or more). Publication bias was assessed using funnel plots where there were at least 10 studies in the meta-analysis. All analyses were performed using R software (metafor and dmetar packages) (23, 24).

## Results

### Search results, quality appraisals

**Figure 1:**
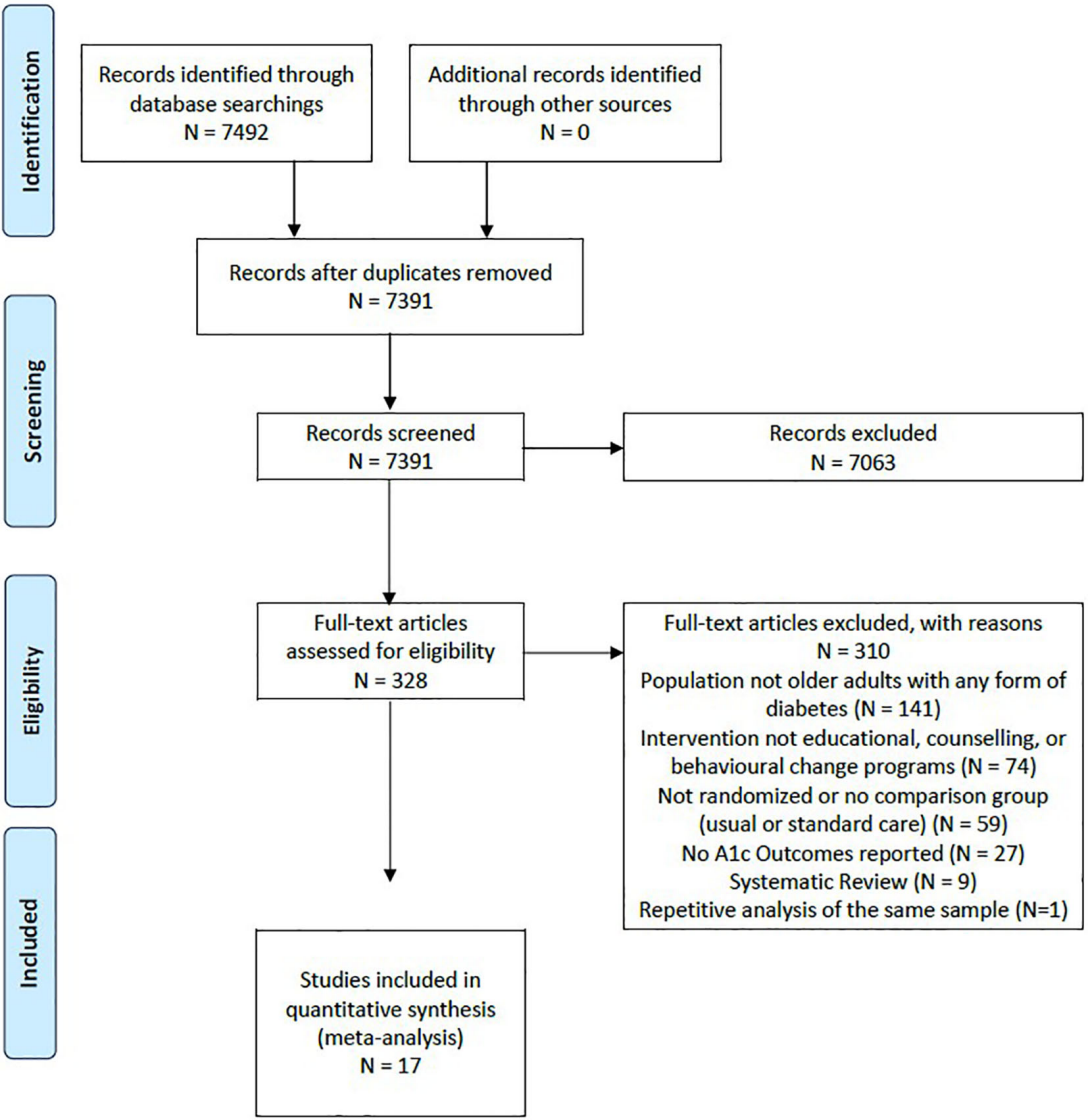
Study flow diagram and selection, according to Preferred Reporting Items for Systematic Reviews and Meta-Analyses.

Our search strategy (**Figure 1**) identified 7391 title and abstract citations for review, with 328 citations reviewed in full text. A total of 17 studies with 21 unique comparison arms were identified that met our inclusion criteria (**Figure 1**). Our included studies had a total sample of 5976 older adults (intervention group: n=3510; usual care group: n=2466). Each intervention arm comprised a self-management approach that fit into the five categories of Chodosh’s framework: 1 study - medical care (25); 3 studies - psychological emphasis (26–28); 4 studies – tailoring (29–32); 4 studies - group setting (33–36); and 5 studies emphasized feedback (37–41). The characteristics of the included studies may be found in **Supplementary Table 3**. The risk of bias was assessed for each study and determined that all of the included studies were considered as unclear for risk of bias (25, 26, 28–41), except for one study which was considered at high risk of bias (27) (**Table 1**).

**Table 1:** Risk of bias assessment (N=17).

Author, Year	SEQUENCE GENERATION	ALLOCATION CONCEALMENT	BLINDING OF PATIENTS/PARTICIPANTS/PERSONNEL	BLINDING OF OUTCOME ASSESSMENT	INCOMPLETE OUTCOME DATA	SELECTIVE REPORTING	OTHER BIAS	Overall Risk of Bias Rating
Seah et al, 2022 (29)	Low	Low	High	Unclear	Unclear	Low	Low	Unclear
Woodard et al, 2022 (33)	Low	Unclear	High	Low	Low	Low	Low	Unclear
Chan et al, 2022 (25)	Low	Low	High	Unclear	Low	Low	Low	Unclear
Poonprapai et al, 2022 (30)	Low	Unclear	Unclear	Unclear	Unclear	Low	Unclear	Unclear
Vasconcelos et al, 2021 (34)	High	Unclear	Unclear	Unclear	Unclear	Low	Low	Unclear
Pai et al, 2021 (37)	Unclear	Low	Unclear	Unclear	Unclear	Low	Low	Unclear
Chen et al, 2021 (38)	Low	Low	Low	Unclear	Low	Low	Low	Unclear
Borba et al, 2020 (35)	Low	Unclear	Unclear	Unclear	High	Low	Unclear	Unclear
Yang et al, 2020 (36)	Low	Unclear	High	Low	Low	Low	Unclear	Unclear
Sun et al, 2019 (39)	Low	Unclear	Unclear	Unclear	Unclear	Low	Unclear	Unclear
De Greef et al, 2011 (27)	Low	Low	High	High	Low	Low	Unclear	High
Munshi et al, 2013 (40)	Low	Unclear	Unclear	Unclear	High	Low	Low	Unclear
Sharifirad et al, 2013 (28)	Unclear	Unclear	Unclear	Unclear	Unclear	Low	Unclear	Unclear
Bond et al, 2007 (26)	Low	Unclear	Unclear	Low	Low	Low	Low	Unclear
Lim et al, 2011 (31)	Low	Unclear	Unclear	Unclear	Low	Low	Unclear	Unclear
Weinstock et al, 2011 (32)	Unclear	Unclear	Unclear	Low	Unclear	Low	Low	Unclear
Braun et al, 2009 (41)	Unclear	High	Unclear	Unclear	Low	Low	Unclear	Unclear

### Glycated hemoglobin A1C

**Figure 2:**
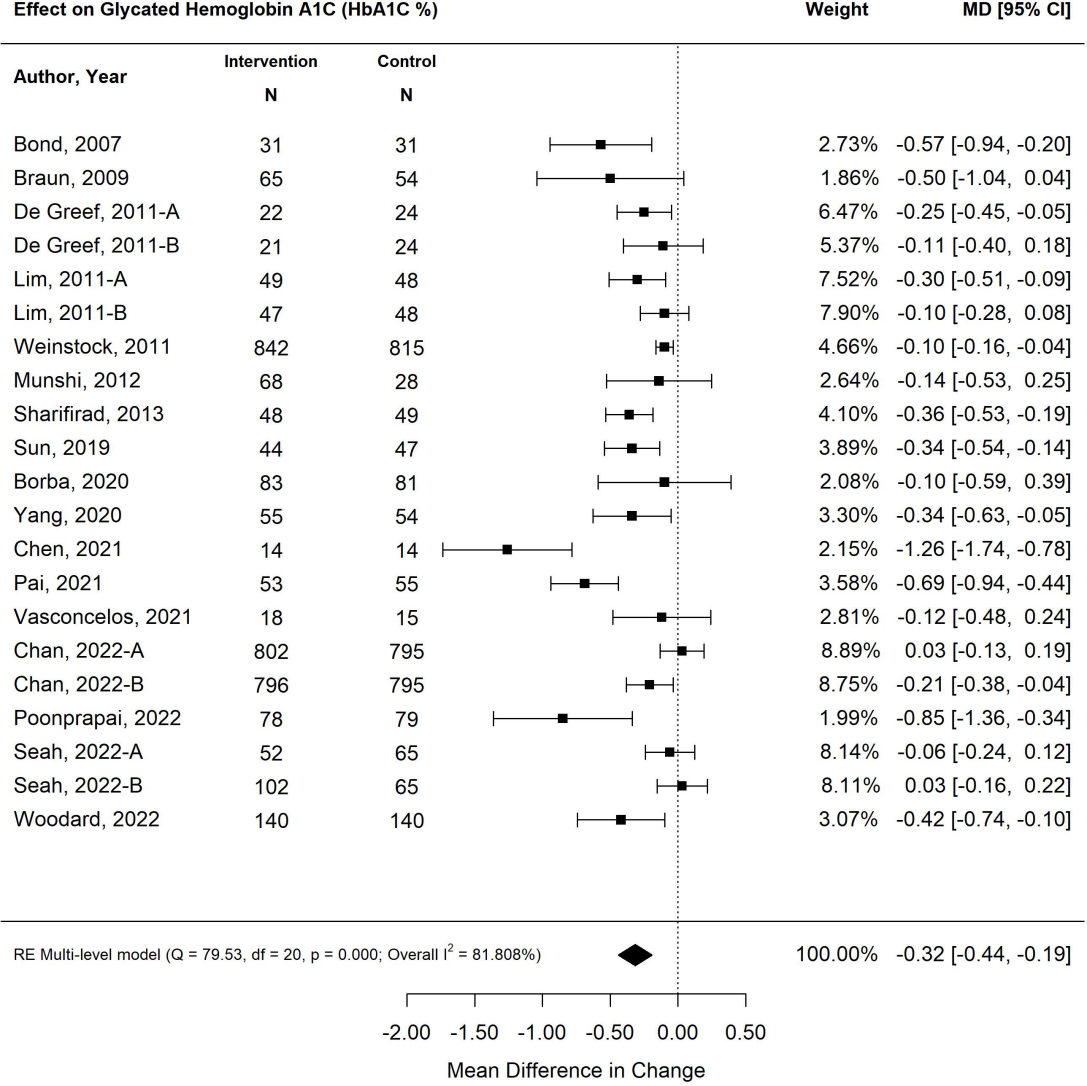
The pooled treatment effect of diabetes self-management program in older adults on glycated hemoglobin A1C (HbA1C).

Overall, the pooled effect of diabetes self-management program interventions for older adults resulted in a statistically significant decrease in HbA1C by 0.32% (95% CI -0.44, -0.19; *p*<0.00) (25–41) (**Figure 2**). The most effective self-management program intervention on HbA1C was the use of feedback (-0.52%; 95% CI -0.68, -0.36; *p*<0.01), followed by psychological emphasis (-0.31%; 95% CI -0.45, -0.16; *p*<0.01); group setting (-0.28%; 95% CI -0.47, -0.09; *p*<0.01) and tailoring (-0.13%; 95% CI -0.22, -0.13; *p*=0.01). Self-management program interventions that emphasized medical care were not statistically significant (-0.09%; 95% CI -0.25, 0.08; *p*=0.03). Self-management interventions that were less than six months in duration resulted in a greater decrease in HbA1C (-0.40%; CI -0.58%, -0.23%; *p*<0.01) than programs that were longer than six months (-0.24%; 95% CI -0.40, -0.7%; *p*<0.01).

### Metabolic outcomes

**Figure 3:**
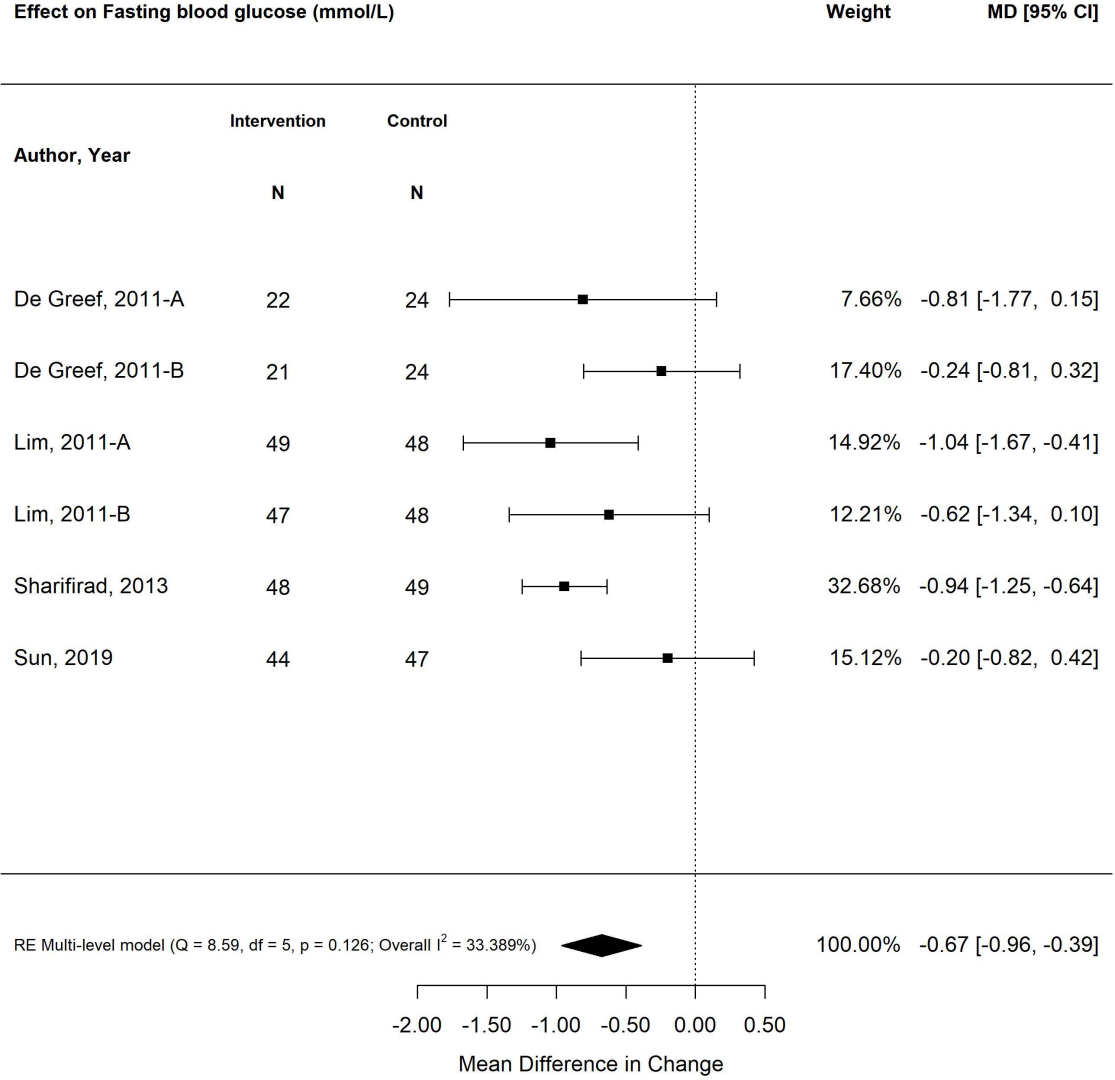
The pooled treatment effect of diabetes self-management programs in older adults on fasting blood glucose (mmol/L).

The pooled treatment effect of diabetes self-management program interventions in older adults on fasting blood glucose (mmol/L) was a reduction in 0.67 mmol/L (95% CI -0.96, -0.39; *p*<0.00) (**Figure 3**) with tailored focused interventions resulting in the greatest decrease (-0.86%; 95% CI -1.35, -0.37; *p*<0.01) (27, 28, 31, 39). Self-management interventions that were less than six months in duration resulted in greater improvements in fasting blood glucose (-0.72 mmol/L; 95% CI -1.09, -0.36; *p*<0.01) than programs longer than six months (-0.62 mmol/L; 95% CI -1.05, -0.19; *p*<0.01).

**Figure 4:**
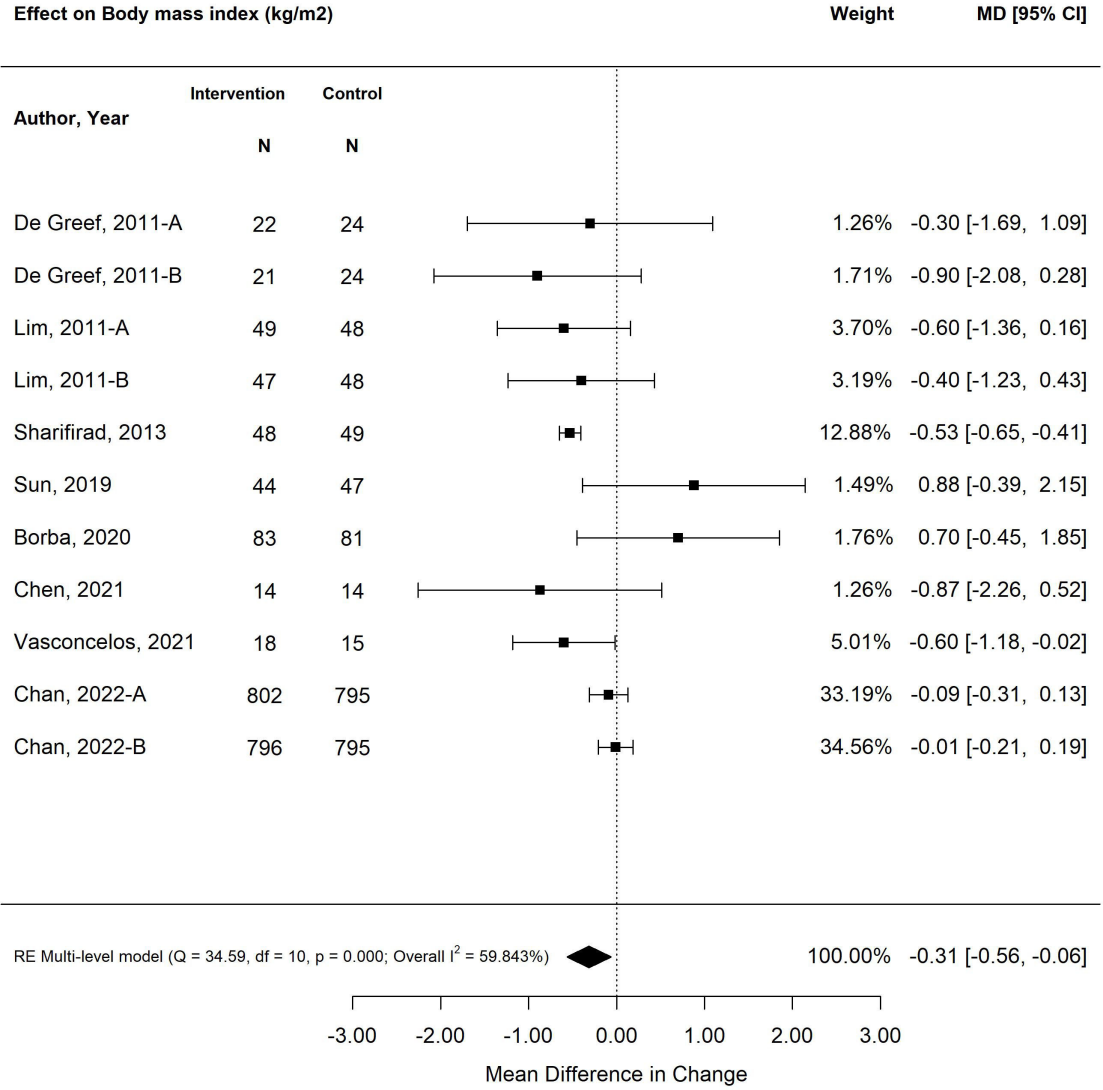
The pooled treatment effect of diabetes self-management programs in older adults on body mass index (BMI).

**Figure 5:**
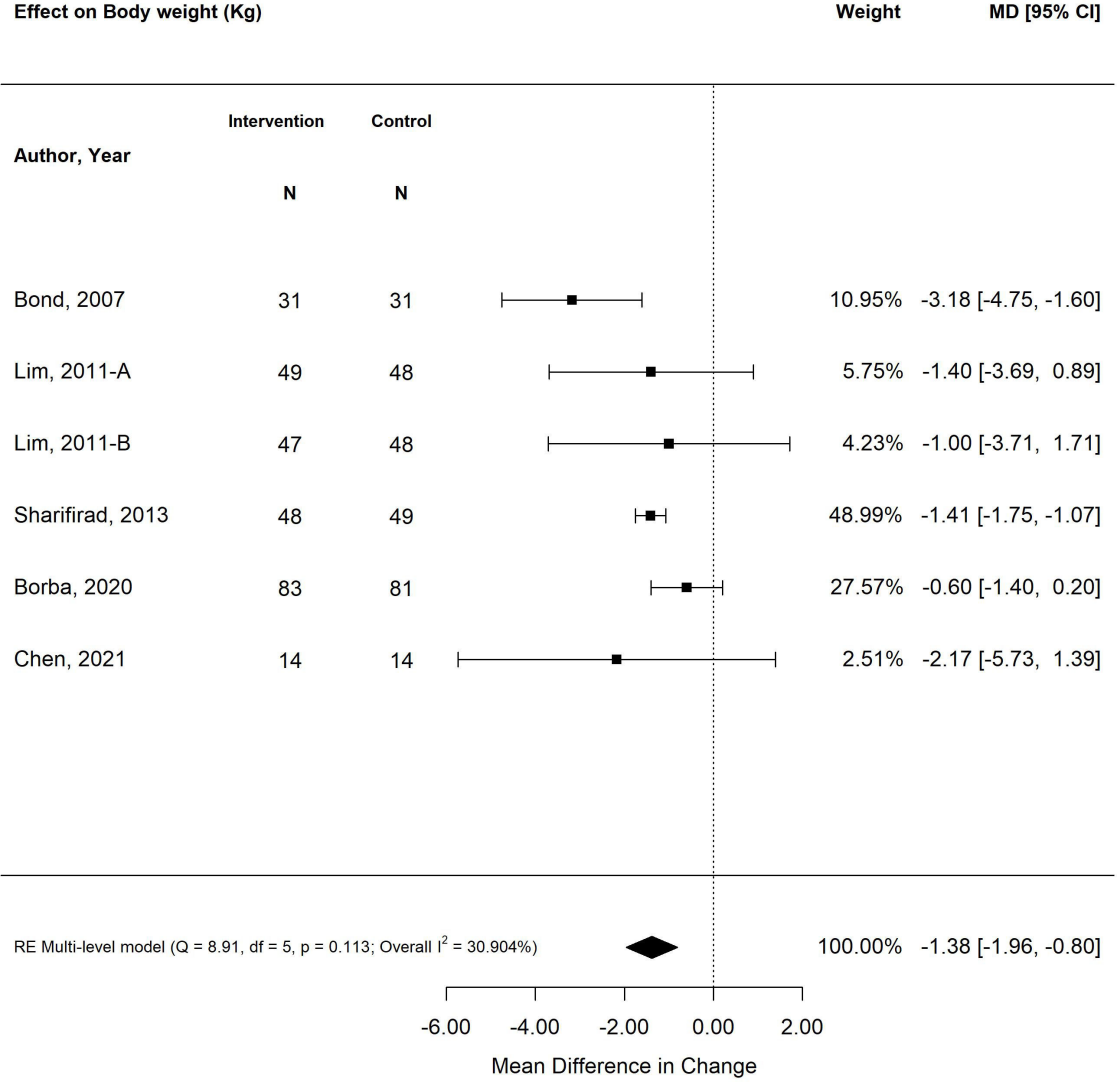
The pooled treatment effect of diabetes self-management programs in older adults on weight (kg).

The pooled treatment effect of diabetes self-management program interventions in older adults on BMI was a reduction of 0.31 (95% CI -0.51, -0.06; *p*<0.01) (**Figure 4**) (25, 27, 28, 31, 34, 35, 38, 39). The self-management program intervention strategy that was most effective in improving BMI was psychological emphasis (-0.53; 95% CI -0.65, -0.41; *p*<0.01). Similarly, self-management program interventions that were less than six months in duration resulted in greater improvement in BMI (-0.54; 95% CI -0.65, -0.42; *p*<0.01) versus programs that were longer than six months (-0.05; 95% CI -0.19, 0.09; *p*=0.46). Similarly, small weight changes were observed in older adults exposed to self-management program interventions, with a decrease in -1.38 kg (95% CI -1.96, -0.80; *p*<0.00) (**Figure 5**) (26, 28, 31, 35, 38). The self-management program intervention strategy that yielded the most weight loss was psychological emphasis (-1.49 kg; 95% CI -1.82, -1.16; *p*<0.01). In accordance with BMI changes, shorter self-management programs (< 6 months) were more effective in weight loss (-1.42 kg; 95% CI -1.76, -1.08; *p*<0.01) versus programs longer than six months (-1.15 kg; 95% CI -1.81, -0.48; *p*<0.01).

The pooled treatment effect of diabetes self-management program interventions in older adults on systolic blood pressure did not result in a statistically significant reduction (-2.20 mmHg; 95% CI-5.49, 1.08; *p*=0.19), regardless of the length of duration of the program (25, 26, 28, 30, 32, 34, 38, 39). Similarly, although the pooled treatment effect of diabetes self-management program interventions in older adults on diastolic blood was statistically significant (-1.48 mm/Hg; 95% CI -2.73, -0.24; *p*=0.02) (25, 26, 28, 30, 32, 34, 38, 39), the reduction is clinically negligible.

The pooled treatment effects of diabetes self-management program interventions in older adults on lipids were varied. There was a small but statistically significant reduction in total cholesterol levels (-0.13 mmol/L; 95% CI -0.20, -0.05; *p*<0.00) (25–27, 31, 32, 38, 39), with tailoring of self-management program interventions as the most effective in reducing total cholesterol (-0.18 mmol/L; 95% CI -0.24, -0.13; *p*<0.01). Similarly, there was a small but statistically significant improvement in triglycerides (-0.12 mmol/L; 95% CI -0.19, -0.05; *p*<0.00) (25, 28, 31, 38, 39), with interventions lasting less than six months (-0.16 mmol/L; 95% CI -0.27, -0.04; *p*<0.01) and offering psychological emphasis yielding the greatest improvement (-0.16 mmol/L; 95% CI -0.29, -0.04; *p*<0.01).

### Patient-reported outcomes (distress, QoL, knowledge, behaviour change, self-efficacy)

**Figure 6:**
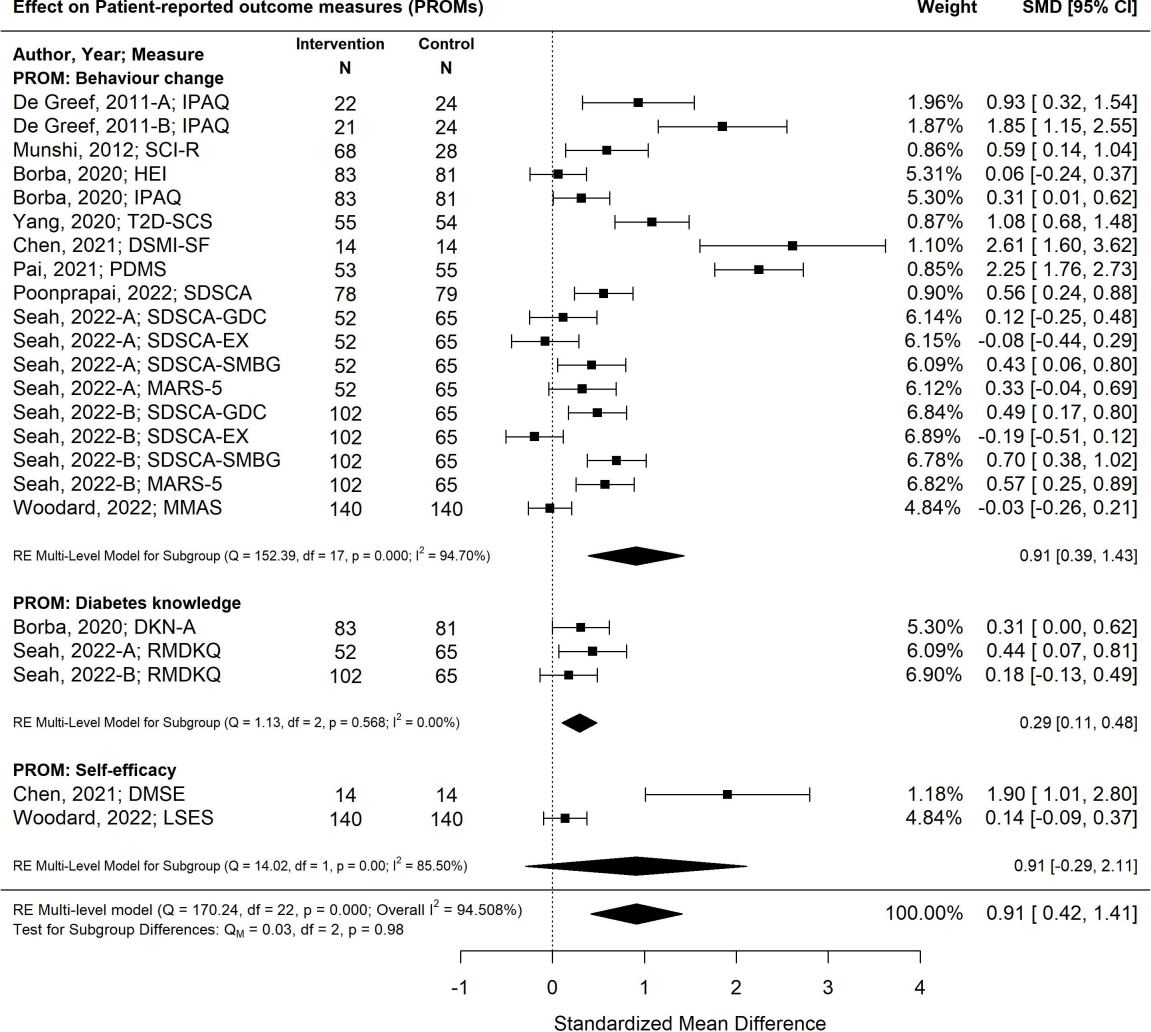
The pooled treatment effect of diabetes self-management programs in older adults on patient-reported outcomes, specifically behaviour change, diabetes knowledge and self-efficacy. IPAQ, International Physical Activity Questionnaire; SCI-R, Self-Care Inventory-R; HEI, Healthy Eating Index; T2D-SCS, Type 2 Diabetes Self-Care Scale; DSMI-SF, Diabetes Self-Management Instrument Short Form; PDMS, Perceived Diabetes Self-Management Scale; SDSCA, Summary of Diabetes Self-Care Activities; SDSCA-GDC, Summary of Diabetes Self Care Activities-Diet; SDSCA-EX, Summary of Diabetes Self-Care Activities-Exercise; SDSCA-SMBG, Summary of Diabetes Self-Care Activities-Self Monitoring Blood Glucose; MARS-5, Medication Adherence Report Scale; MMAS, Morisky Medication Adherence Scale; DKN-A, Diabetes Knowledge Scale; RMDKQ, Revised Michigan Diabetes Knowledge Questionnaire; DMSE, Diabetes Self-Management Instrument; LSES, Lorig Self-efficacy Scale.

The pooled effect of diabetes self-management program interventions for older adults resulted in statistically significant improvements in the standardized mean differences in patient-reported outcomes measures for behaviour change, diabetes knowledge and self-efficacy (-0.91 SMD; 95% CI -042, 1.41; *p*<0.00) (27, 29, 30, 33, 35–38, 40) (**Figure 6**). The most effective self-management program intervention strategy associated with the greatest improvements in patient-reported outcomes was feedback (1.64; 95% CI 1.08, 2.21; *p*<0.01). Self-management program interventions that were less than six months were more effective (1.06; 95% CI 0.44, 1.69; *p*<0.01) versus programs that were longer than six months (0.60; 95% CI -0.26, 1.47; *p*=0.17).

## Discussion

Diabetes self-management programs for older adults continue to evolve, particularly for older adults living with multimorbidity. Our updated review continues to demonstrate the value of self-management programs on clinical and patient-reported outcomes by synthesizing the available evidence and identifying the most effective self-management approaches to use based on the type of outcome. Overall, diabetes self-management program interventions for older adults were found to reduce HbA1C by -0.32%, a statistically significant and clinically meaningful improvement, particularly as self-management programs are offered in conjunction with pharmacotherapy (9–11, 13). Notably, providing self-management programs with the addition of feedback, as defined by Chodosh’s framework, offered an improvement of 0.52%, which is similar to that of some pharmacotherapy agents. Self-management program interventions that offered feedback were also found to be most effective in improving patient-reported outcomes, specifically behaviour change (SMD 0.91; 95% CI 0.39, 1.43). Finally, self-management program interventions that were less than six months in duration were associated with improved clinical and patient-reported outcomes. This finding may be insightful for the design, implementation, scale up and sustainability of future self-management programs.

### Strengths

The updated literature reflected all of the possible categories of Chodosh’s framework for self-management programs for older adults, highlighting an evolution and distribution of self-management approaches (13). The literature also reflected a broader reflection of outcomes, including patient-centred outcomes, such as behaviour change. Of note, many outcomes found some small but significant improvements that are noteworthy as self-management programs are often in conjunction with pharmacotherapy and comorbid conditions that require similar behaviors management, nutrition, and physical activity approaches.

### Limitations

Our updated review included peer-reviewed literature published in English from November 2013 to July 2023. We recognize that literature may have been missed; however, we were able to apply the same search strategy to five databases yielding a large pool of citations. Unfortunately, concerning the representation of literature within the categories of Chodosh’s framework, medical care in self-management programs continues to be minimally represented in the literature to date. We found the literature to be susceptible to bias as most of the studies were deemed to have an unclear risk of bias due to a lack of reporting (14).

### Conclusion

Our review builds on the findings of our previous review (12) as it provides valuable insight into new literature that has emerged on this topic, specifically to understand the impact of specific self-management programs on clinical and patient-reported outcomes for older adults living with diabetes. The findings help to better understand the growing body of literature regarding older adults receiving self-management education and support to meet their complex social, cognitive, medical, functional, and psychological needs (2–7). The findings of this review highlight the need for customization of self-management programs to optimize clinical and patient-centred outcomes, specifically noting that feedback strategies and programs of shorter duration (< six months) were most effective. Future research should explore the need for combination strategies (e.g. feedback and psychological emphasis) to address the complex needs of older adults living with diabetes and multimorbidity. Additionally, future research may also consider cost analyses of self-management programs in supporting older adults to remain out of the hospital, decrease the risks and impact of complications, and age in place. Just as no two people are the same, the findings of this updated review demonstrate that to improve different outcomes, customized self-management strategies for older adults should be employed.

## Data availability statement

The original contributions presented in the study are included in the article/**Supplementary Material**. Further inquiries can be directed to the corresponding author.

## Author contributions

PA: Writing – original draft, Writing – review & editing. MJ: Writing – review & editing. SK: Writing – review & editing. DF: Writing – review & editing. MA: Writing – original draft, Writing – review & editing. DS: Writing – original draft, Writing – review & editing.
