# Supplementary material for: The effects of diabetes self-management programs on clinical and patient reported outcomes in older adults: a systematic review and meta-analysis
Source: Front Clin Diabetes Healthc. 2024 Jun 17;5:1348104. doi: 10.3389/fcdhc.2024.1348104 (PMC11215190; doi:10.3389/fcdhc.2024.1348104)
Supplement: Supplementary file 1 [file DataSheet_1.docx]

Supplementary Material

# Supplementary Tables

Supplemental Table 1: Search Strategy

Medline-OVID

July 20, 2023

1. (elderly or older or aged or seniors or geriatric? or vetrans).ti.

2. *Long-Term Care/

3. nursing homes.ti.

4. geriatric?.jn.

5. medicare.ti.

6. aged/ or Geriatrics/

7. or/1-6

8. *diabetes mellitus/ or exp *diabetes mellitus, type 1/ or exp *diabetes mellitus, type 2/

9. ((type 2 or type II or non-insulin dependent) adj3 diabet*).ti.

10. (MODY or NIDDM or T2DM).ti.

11. ((type1 or type 1 or insulin dependent or IDDM) adj3 diabet*).ti.

12. ((diabetes or diabetic) not gestation*).ti.

13. or/8-12

14. Patient Education as Topic/

15. self care/

16. self management.tw.

17. self efficacy/

18. motivational interviewing.tw.

19. health coaching.tw.

20. solution focused therapy.tw.

21. support.ti.

22. *health education/

23. *Education/

24. ((self or home) adj3 (monitoring or testing)).tw.

25. *patient participation/

26. Blood Glucose Self-Monitoring/

27. (smbg or dsme).tw.

28. empowerment.tw.

29. Health Educators/

30. family involvement.ti.

31. or/14-30

32. *Blood Glucose Self-Monitoring/

33. (smbg or dsme).tw. or ((diabetes or diabetic) and education).ti.

34. 32 or 33

35. (pediatric* or paediatric* or child* or adolescent?).jn.

36. (pediatric* or paediatric* or child* or adolescent? or youth? or teenager? or teen?).ti.

37. (gestation* or pregnan*).ti.

38. 31 or 34

39. 7 and 13 and 38

40. 35 or 36 or 37

41. 39 not 40

42. limit 41 to (english language and humans)

43. limit 42 to (comment or editorial or letter or newspaper article)

44. 42 not 43

45. limit 44 to yr="2003 -Current"

Embase-OVID

July 20, 2023

1. aged/ and age/

2. (elderly or older or aged or seniors or geriatric? or vetrans).ti.

3. *nursing home/ or *nursing home patient/

4. medicare.ti.

5. exp *geriatrics/

6. geriatric?.jn.

7. or/1-6

8. diabetes mellitus/ or exp *diabetic angiopathy/ or *insulin dependent diabetes mellitus/ or *maturity onset diabetes mellitus/ or *non insulin dependent diabetes mellitus/

9. ((type1 or type 1 or insulin dependent or IDDM) adj3 diabet*).ti.

10. ((diabetes or diabetic) not gestation*).ti.

11. or/8-10

12. *health education/ or patient education/

13. exp self care/

14. self management.tw.

15. self efficacy.tw.

16. motivational interviewing/

17. *patient counseling/

18. motivational interviewing.tw.

19. health coaching.mp.

20. solution focused therapy.mp.

21. ((self or home) adj3 (monitoring or testing)).tw.

22. *EDUCATION/ or *EDUCATION PROGRAM/

23. *patient participation/

24. empowerment/

25. support.ti.

26. health educator/

27. family involvement.ti.

28. educational model/

29. health education/

30. diabetes education/ or diabetes educator/

31. (smbg or dsme).tw. or ((diabetes or diabetic) and education).ti.

32. *blood glucose monitoring/

33. *self monitoring/

34. 32 and 33

35. or/12-29

36. 30 or 31 or 34

37. 7 and 36

38. 7 and 11 and 35

39. 37 or 38

40. limit 39 to (human and english language)

41. limit 40 to (book or book series or editorial or letter or note)

42. 40 not 41

43. limit 42 to (conference abstract or conference paper or conference proceeding or "conference review")

44. 42 not 43

45. limit 44 to yr="2003 -Current"

Cochrane Controlled Trials Registry-OVID

July 20, 2023

1. (elderly or older or aged or seniors or geriatric? or vetrans).ti.

2. *Long-Term Care/

3. nursing homes.ti.

4. geriatric?.jn.

5. medicare.ti.

6. aged/ or Geriatrics/

7. or/1-6

8. *diabetes mellitus/ or exp *diabetes mellitus, type 1/ or exp *diabetes mellitus, type 2/

9. ((type 2 or type II or non-insulin dependent) adj3 diabet*).ti.

10. (MODY or NIDDM or T2DM).ti.

11. ((type1 or type 1 or insulin dependent or IDDM) adj3 diabet*).ti.

12. ((diabetes or diabetic) not gestation*).ti.

13. or/8-12

14. Patient Education as Topic/

15. self care/

16. self management.tw.

17. self efficacy/

18. motivational interviewing.tw.

19. health coaching.tw.

20. solution focused therapy.tw.

21. support.ti.

22. *health education/

23. *Education/

24. ((self or home) adj3 (monitoring or testing)).tw.

25. *patient participation/

26. Blood Glucose Self-Monitoring/

27. (smbg or dsme).tw.

28. empowerment.tw.

29. Health Educators/

30. family involvement.ti.

31. or/14-30

32. *Blood Glucose Self-Monitoring/

33. (smbg or dsme).tw. or ((diabetes or diabetic) and education).ti.

34. 32 or 33

35. (pediatric* or paediatric* or child* or adolescent?).jn.

36. (pediatric* or paediatric* or child* or adolescent? or youth? or teenager? or teen?).ti.

37. (gestation* or pregnan*).ti.

38. 31 or 34

39. 7 and 13 and 38

40. 35 or 36 or 37

41. 39 not 40

42. limit 41 to yr="2003 -Current"

PsycINFO-OVID

July 20, 2023

1. (elderly or older or aged or seniors or geriatric? or vetrans).ti.

2. aging/ or geriatrics/ or gerontology/

3. geriatric patients/

4. exp Long Term Care/

5. geriatric*.mp.

6. medicare.ti.

7. or/1-6

8. exp diabetes/

9. ((type 2 or type II or non-insulin dependent) adj3 diabet*).ti.

10. (MODY or NIDDM or T2DM).ti.

11. ((diabetes or diabetic) not gestation*).ti.

12. or/8-11

13. exp Self Management/ or exp Self Monitoring/

14. exp Health Education/ or exp Educational Personnel/ or exp Training/

15. client education/

16. health education/

17. self care skills/

18. exp Self Efficacy/

19. self management.tw.

20. health coaching.tw.

21. ((self or home) adj3 (monitoring or testing)).tw.

22. (smbg or dsme).tw. or ((diabetes or diabetic) and education).ti.

23. empowerment.tw.

24. family involvement.ti.

25. or/13-24

26. 7 and 12 and 25

27. limit 26 to (human and english language)

28. limit 27 to (chapter or "column/opinion" or "comment/reply" or editorial or letter or obituary or review-book)

29. 27 not 28

30. "diabetes education".id.

31. diabetes education.tw.

32. 30 or 31

33. 7 and 32

34. (elderly or older or aged or seniors or geriatric? or vetrans).ti.

35. aging/ or geriatrics/ or gerontology/ or elder care/ or aging in place/

36. geriatric patients/

37. exp Long Term Care/

38. geriatric*.mp.

39. medicare.ti.

40. or/34-39

41. exp diabetes/

42. ((type 2 or type II or non-insulin dependent) adj3 diabet*).ti.

43. (MODY or NIDDM or T2DM).ti.

44. ((diabetes or diabetic) not gestation*).ti.

45. or/41-44

46. exp Self Management/ or exp Self Monitoring/

47. exp Health Education/ or exp Educational Personnel/ or exp Training/

48. client education/

49. health education/

50. self care skills/

51. exp Self Efficacy/

52. self management.tw.

53. health coaching.tw.

54. ((self or home) adj3 (monitoring or testing)).tw.

55. (smbg or dsme).tw. or ((diabetes or diabetic) and education).ti.

56. empowerment.tw.

57. family involvement.ti.

58. or/46-57

59. 40 and 45 and 58

60. "diabetes education".id.

61. diabetes education.tw.

62. 60 or 61

63. 40 and 62

64. 59 or 63

65. limit 64 to (human and english language)

66. limit 65 to (chapter or "column/opinion" or "comment/reply" or editorial or letter or obituary or review-book)

67. 65 not 66

68. limit 67 to (("0200 book" or "0240 authored book" or "0280 edited book" or "0300 encyclopedia" or "0400 dissertation abstract") and (abstract collection or dissertation))

69. 67 not 68

70. limit 69 to yr="2003 -Current"

CINAHL-EBSCO

July 20, 2023

| S20 | ( S17 OR S18 ) NOT S19 | Limiters - Language: English  Search modes - Boolean/Phrase |
| --- | --- | --- |
| S19 | S17 OR S18 | Limiters - Publication Type: Book, Book Chapter, Book Review, Commentary, Editorial, Letter  Search modes - Boolean/Phrase |
| S18 | S5 AND S8 AND S15 | Search modes - Boolean/Phrase |
| S17 | S5 AND S16 | Search modes - Boolean/Phrase |
| S16 | (MH "Diabetes Education") OR (MH "American Association of Diabetes Educators") OR (MH "Diabetes Educators") | Search modes - Boolean/Phrase |
| S15 | S9 OR S10 OR S11 OR S12 OR S13 OR S14 | Search modes - Boolean/Phrase |
| S14 | (MH "Models, Educational") | Search modes - Boolean/Phrase |
| S13 | (MH "Consumer Participation") | Search modes - Boolean/Phrase |
| S12 | TX ((self or home) N3 (monitoring or testing)) | Search modes - Boolean/Phrase |
| S11 | TX health coaching | Search modes - Boolean/Phrase |
| S10 | (MH "Patient Education") OR (MH "Health Education") | Search modes - Boolean/Phrase |
| S9 | (MH "Self Care") OR (MH "Self Administration") | Search modes - Boolean/Phrase |
| S8 | S6 OR S7 | Search modes - Boolean/Phrase |
| S7 | TI ( diabetes or diabetic ) NOT TI gestation* | Search modes - Boolean/Phrase |
| S6 | (MH "Diabetes Mellitus") OR (MH "Diabetes Mellitus, Type 1") OR (MH "Diabetes Mellitus, Type 2") OR (MH "Diabetic Patients") | Search modes - Boolean/Phrase |
| S5 | S1 OR S2 OR S3 OR S4 | Search modes - Boolean/Phrase |
| S4 | SO geriatric* | Search modes - Boolean/Phrase |
| S3 | (MH "American Geriatrics Society") OR (MH "Geriatrics") OR (MH "Geriatric Functional Assessment") OR (MH "Geriatric Psychiatry") OR (MH "Rehabilitation, Geriatric") OR (MH "Gerontologic Nurse Practitioners") OR (MH "Gerontologic Care") OR (MH "Gerontologic Nursing") | Search modes - Boolean/Phrase |
| S2 | TI (elderly or older or aged or seniors or geriatric? or vetrans) | Search modes - Boolean/Phrase |
| S1 | (MH "Aged+") OR (MH "Aged, 80 and Over") OR (MH "Health Services for the Aged") | Search modes - Boolean/Phrase |

Supplemental Table 2: PRISMA Checklist

| **Section and Topic** | **Item #** | **Checklist item** | **Location where item is reported** |
| --- | --- | --- | --- |
| **TITLE** | | |  |
| Title | 1 | Identify the report as a systematic review. | p.1 |
| **ABSTRACT** | | |  |
| Abstract | 2 | See the PRISMA 2020 for Abstracts checklist. | p.2 |
| **INTRODUCTION** | | |  |
| Rationale | 3 | Describe the rationale for the review in the context of existing knowledge. | p.3 |
| Objectives | 4 | Provide an explicit statement of the objective(s) or question(s) the review addresses. | p.3 |
| **METHODS** | | |  |
| Eligibility criteria | 5 | Specify the inclusion and exclusion criteria for the review and how studies were grouped for the syntheses. | p.4 |
| Information sources | 6 | Specify all databases, registers, websites, organisations, reference lists and other sources searched or consulted to identify studies. Specify the date when each source was last searched or consulted. | p.4 |
| Search strategy | 7 | Present the full search strategies for all databases, registers and websites, including any filters and limits used. | Supplemental File 2 |
| Selection process | 8 | Specify the methods used to decide whether a study met the inclusion criteria of the review, including how many reviewers screened each record and each report retrieved, whether they worked independently, and if applicable, details of automation tools used in the process. | p.4 |
| Data collection process | 9 | Specify the methods used to collect data from reports, including how many reviewers collected data from each report, whether they worked independently, any processes for obtaining or confirming data from study investigators, and if applicable, details of automation tools used in the process. | p.4 |
| Data items | 10a | List and define all outcomes for which data were sought. Specify whether all results that were compatible with each outcome domain in each study were sought (e.g. for all measures, time points, analyses), and if not, the methods used to decide which results to collect. | p.4 |
|  | 10b | List and define all other variables for which data were sought (e.g. participant and intervention characteristics, funding sources). Describe any assumptions made about any missing or unclear information. | p.4 |
| Study risk of bias assessment | 11 | Specify the methods used to assess risk of bias in the included studies, including details of the tool(s) used, how many reviewers assessed each study and whether they worked independently, and if applicable, details of automation tools used in the process. | p.4 |
| Effect measures | 12 | Specify for each outcome the effect measure(s) (e.g. risk ratio, mean difference) used in the synthesis or presentation of results. | p.4 & 5 |
| Synthesis methods | 13a | Describe the processes used to decide which studies were eligible for each synthesis (e.g. tabulating the study intervention characteristics and comparing against the planned groups for each synthesis (item #5)). | p. 4 & 5 |
|  | 13b | Describe any methods required to prepare the data for presentation or synthesis, such as handling of missing summary statistics, or data conversions. | p. 4 & 5 |
|  | 13c | Describe any methods used to tabulate or visually display results of individual studies and syntheses. | p.4 |
|  | 13d | Describe any methods used to synthesize results and provide a rationale for the choice(s). If meta-analysis was performed, describe the model(s), method(s) to identify the presence and extent of statistical heterogeneity, and software package(s) used. | p. 4 & 5 |
|  | 13e | Describe any methods used to explore possible causes of heterogeneity among study results (e.g. subgroup analysis, meta-regression). | p. 4 & 5 |
|  | 13f | Describe any sensitivity analyses conducted to assess robustness of the synthesized results. | p.4 & 5 |
| Reporting bias assessment | 14 | Describe any methods used to assess risk of bias due to missing results in a synthesis (arising from reporting biases). | p.4 & 5 |
| Certainty assessment | 15 | Describe any methods used to assess certainty (or confidence) in the body of evidence for an outcome. | p. 4 & 5 |
| **RESULTS** | | |  |
| Study selection | 16a | Describe the results of the search and selection process, from the number of records identified in the search to the number of studies included in the review, ideally using a flow diagram. | p.5 |
|  | 16b | Cite studies that might appear to meet the inclusion criteria, but which were excluded, and explain why they were excluded. | N/A |
| Study characteristics | 17 | Cite each included study and present its characteristics. | p. 5, 6, Supplemental Table 1 |
| Risk of bias in studies | 18 | Present assessments of risk of bias for each included study. | p.5 |
| Results of individual studies | 19 | For all outcomes, present, for each study: (a) summary statistics for each group (where appropriate) and (b) an effect estimate and its precision (e.g. confidence/credible interval), ideally using structured tables or plots. | p. 5, 6, Figures 2-6 |
| Results of syntheses | 20a | For each synthesis, briefly summarise the characteristics and risk of bias among contributing studies. | N/A |
|  | 20b | Present results of all statistical syntheses conducted. If meta-analysis was done, present for each the summary estimate and its precision (e.g. confidence/credible interval) and measures of statistical heterogeneity. If comparing groups, describe the direction of the effect. | p.5 & 6 |
|  | 20c | Present results of all investigations of possible causes of heterogeneity among study results. | p.5 |
|  | 20d | Present results of all sensitivity analyses conducted to assess the robustness of the synthesized results. | N/A |
| Reporting biases | 21 | Present assessments of risk of bias due to missing results (arising from reporting biases) for each synthesis assessed. | N/A |
| Certainty of evidence | 22 | Present assessments of certainty (or confidence) in the body of evidence for each outcome assessed. | p.5 & 6, Figures 2-6 |
| **DISCUSSION** | | |  |
| Discussion | 23a | Provide a general interpretation of the results in the context of other evidence. | p.7 |
|  | 23b | Discuss any limitations of the evidence included in the review. | p.7 |
|  | 23c | Discuss any limitations of the review processes used. | p.7 |
|  | 23d | Discuss implications of the results for practice, policy, and future research. | p.7 |
| **OTHER INFORMATION** | | |  |
| Registration and protocol | 24a | Provide registration information for the review, including register name and registration number, or state that the review was not registered. | N/A |
|  | 24b | Indicate where the review protocol can be accessed, or state that a protocol was not prepared. | N/A |
|  | 24c | Describe and explain any amendments to information provided at registration or in the protocol. | N/A |
| Support | 25 | Describe sources of financial or non-financial support for the review, and the role of the funders or sponsors in the review. | p.8 |
| Competing interests | 26 | Declare any competing interests of review authors. | p.8 - confirm |
| Availability of data, code and other materials | 27 | Report which of the following are publicly available and where they can be found: template data collection forms; data extracted from included studies; data used for all analyses; analytic code; any other materials used in the review. | N/A |

Supplemental Table 3: Characteristics of included studies (N=17).

| Authors, Year | Seah et al, 2022 |
| --- | --- |
| Purpose/Objective | To investigate the effectiveness of a community-based intervention on improving knowledge about diabetes, self-care behaviors, and glycemic control among older adults with type 2 diabetes mellitus (T2DM) in Singapore, a country in Asia with a high prevalence of diabetes. |
| Study Design | Randomized controlled trial |
| Country/Setting | Singapore/30 community sites including senior activity centers and community clubs |
| Study Duration | 12 months |
| Sample Size | O: 257; SCOPE-DM: 61, SCOPE-DM with glucometer and accessories: 117; C: 79 |
| Inclusion/Exclusion Criteria | Inclusion: Community-dwelling individuals with T2DM between the ages of 55 and 99 years were included. The diagnosis of T2DM was made prior by a medical practitioner.  Exclusion: Type 1 diabetes, mentally incapacitated, or unable to communicate in English, Mandarin, or Malay. |
| Intervention Description/Delivery | Description: The SCOPE-DM program was a 12-session intervention program conducted by trained diabetes nurse educators between March 2019 and October 2020 that aimed to provide motivation and enable capability for managing diabetes. Each session was designed to teach knowledge and practical skills in diabetes self-care with psychological techniques for behavioral change. The emphasis was on empowering individuals to make choices and lifestyle changes in line with psychological techniques for behavioral change such as problem solving, goal setting, and feedback. The content and delivery format were designed using the principles of motivational interviewing (MI).  Delivery: in-person group-based |
| Control Description | Received routine care from their health care providers. |
| Follow Up (%) | SCOPE-DM: 85; SCOPE-DM with glucometer and accessories: 87; C: 83 |
| Mean Age (SD) | O: 71.6 (NR) |
| Male (%) | SCOPE-DM :5; SCOPE-DM With Glucometer and Accessories: 12; C: 16 |
| Race (%) | NR |
| SES/Education | Education background: n (%)  No formal education/primary level: SCOPE-DM: 37 (14), SCOPE-DM With Glucometer and Accessories: 59 (23) ; C: 36 (14)  Secondary: SCOPE-DM: 16 (6), SCOPE-DM With Glucometer and Accessories: 46 (18); C: 17 (6)  Postsecondary (vocational/junior college/polytechnic/university and above): SCOPE-DM: 8 (3), SCOPE-DM With Glucometer and Accessories: 12 (5); C: 26 (10) |
| Type/Duration of Diabetes | Type: T2DM  Duration: NR |
| Diabetes Treatment | NR |
| Comorbidities | NR |
| Outcome(s) Description | Knowledge about diabetes: Revised Michigan Diabetes Knowledge Questionnaire (RMDKQ); Diabetes self-care behaviors: Revised Summary of Diabetes Self-Care Activities (RSDSCA); Medication Adherence: Medication Adherence Report Scale (MARS-5); A1C |
| Type of Analysis | Descriptive analyses were conducted for all outcome variables at baseline and 3-month and 6-month follow-up. Between-group inferential analyses were conducted using linear mixed models. |
| Tools for Outcome Assessment | Revised Michigan Diabetes Knowledge Questionnaire (RMDKQ), Revised Summary of Diabetes Self-Care Activities (RSDSCA), Medication Adherence Report Scale (MARS-5) |
| Frequency of Outcome Assessment | Baseline, 3-month and 6-month follow-up |
| Outcome Results | Knowledge, behavioral, clinical |
| Study Findings | The community-based intervention should be extended to more older adults with T2DM in the community. Glucometers and accessories could be provided at subsidized rates or be made free contingent on older adults’ income status to overcome the barrier of performing SMBG. |
| Chadosh Framework Category | Tailoring |

| Authors, Year | Woodard et al, 2022 |
| --- | --- |
| Purpose/Objective | To evaluate the implementation and effectiveness of Empowering Patients in Chronic Care (EPICC), an evidence-based intervention to improve diabetes-associated distress and hemoglobin A1c (HbA1c) levels after the intervention and after 6-month maintenance. |
| Study Design | Randomized clinical trial |
| Country/Setting | USA/Veterans Affairs clinics |
| Study Duration | 10 months |
| Inclusion/Exclusion Criteria | Inclusion: Uncontrolled type 2 diabetes with a mean HbA1c level >8.0% in the prior 6 months who received primary care at participating clinics in the previous year.  Exclusion: Hearing or vision impairment; active substance use disorder (within 1 year); active bipolar or psychotic disorder; dementia; severe hypoglycemia (defined as a glucagon prescription); limited life expectancy (identified using a validated algorithm); or death; baseline HbA1c level was less than 7.5% or if they were unwilling to participate in regular group sessions. |
| Sample Size | O: 280; I: 140; C: 140 |
| Intervention Description/Delivery | Description: EPICC participants attended 6 bimonthly group sessions (duration of approximately 1 hour) based on collaborative goal setting and motivational interviewing theory during a 3-month period. A 3-hour training workshop prepared health care professionals (physicians, nurse educators, nurse practitioners, pharmacists, dietitians, and psychologists) to lead sessions and conduct 10-minute individual sessions immediately following group sessions with each participant. During the individual sessions, participants discussed their personal concerns and questions, set and adjusted collaborative goals, and reviewed changes to medications or other recommended care.  Delivery: in-person |
| Control Description | Routine care that included diabetes management educational materials, nutrition counseling, medication management or weight loss support, a list of self-management resources routinely offered at their site (eg, traditional diabetes education), and communication with their primary care clinician indicating the desire for additional diabetes resources. |
| Follow Up (%) | I: 83; C: 81 |
| Mean Age (SD) | O: 67.2 (8.4); I: 67.4 (8.6); C: 66.9 (8.3) |
| Male (%) | I: 94; C: 95 |
| Race (%) | Non-Hispanic White: I: 50; C: 46 |
| SES/Education | Educational attainment, n (%): I; C  High school graduate or less: 37 (26); 33 (24)  Some college or more: 103 (74); 107 (74)  Annual income ($), n (%), I; C  <20000: 41 (31); 39 (31)  20000-39999: 38 (29); 37 (29)  ≥40000: 52 (40); 51 (40) |
| Type/Duration of Diabetes | Type: T2DM  Duration: NR |
| Diabetes Treatment | NR |
| Comorbidities | NR |
| Outcome(s) Description | Primary: HbA1c  Secondary: diabetes-associated distress, adherence, and self-efficacy |
| Type of Analysis | RE-AIM (reach, effectiveness, adoption, implementation, and maintenance)  χ2 tests and independent samples t tests |
| Tools for Outcome Assessment | HbA1c: ion-exchange liquid chromatography  Diabetes Distress: Diabetes Distress Scale (DDS)  Medication Adherence: Morisky Medication Adherence Scale  Self-efficacy: Lorig Self-efficacy Scale |
| Frequency of Outcome Assessment | Baseline, 4 months after enrollment, 10 months after enrollment |
| Outcome Results | Clinical, knowledge, behavioral |
| Study Findings | A patient-empowerment approach using longitudinal collaborative goal setting and motivational interviewing is feasible in primary care. Improvements in HbA1c levels after the intervention were not sustained after maintenance. Modest improvements in diabetes-associated distress after the intervention were sustained after maintenance. Innovations to expand reach (eg, telemedicine-enabled shared appointments) and sustainability are needed. |
| Chadosh Framework Category | Group |

| Authors, Year | Chan et al, 2022 |
| --- | --- |
| Purpose/Objective | To evaluate the effect of the Joint Asia Diabetes Evaluation (JADE) web portal, nurse reminders, and team-based care on multiple risk factors in patients with DKD. |
| Study Design | Randomized clinical trial |
| Country/Setting | China, Hong Kong, Malaysia, Philippines, South Korea, Taiwan, Thailand, Vietnam/13 hospital-based diabetes centers |
| Study Duration | 12 months |
| Inclusion/Exclusion Criteria | Inclusion: Type 2 diabetes, which was defined as nonketotic presentation or no insulin requirement within 1 year of diagnosis; DKD either an eGFR of less than 60 mL/min/1.73m2 or serum creatinine with a 30% or more upper reference limit  Exclusion: eGFR of less than 15 mL/min/1.73m2, the need for kidney replacement therapy, inability to give consent, and life-threatening illnesses or conditions that were considered unsuitable by the investigators at each site |
| Sample Size | O: 2393; Empowered care: 802, Team-based empowered care: 796; C: 795 |
| Intervention Description/Delivery | Description: The JADE web portal consists of templates that guide the comprehensive assessment of eye, feet, blood, and urine and documentation of demographic characteristics, socioeconomic status, lifestyle, medical history, physical assessments, laboratory measurements, and medications using a standardized case report form. The portal incorporates validated risk equations for risk stratification and issues a personalized report with automated decision support for patients and physicians. Those in the empowered care group received a personalized report with a nurse explanation during a face-to-face visit and telephone reminders on adherence to clinic visits, medication, and self-management from a nurse every 3 months. In addition to these procedures, those in the team-based empowered care group attended a clinic visit every 3 months that was managed by a team of 1 nurse and 1 physician.  Delivery: hybrid: in-person and telephone |
| Control Description | Usual care |
| Follow Up (%) | Empowered care: 70; Team-based empowered care: 64; C: 82 |
| Mean Age (SD) | O: 67.7 (9.8); Empowered care: 67.5 (10.2), Team-based empowered care: 67.5 (9.4); C: 67.9 (9.9) |
| Male (%) | Empowered care: 52, Team-based empowered care: 54; C: 54 |
| Race (%) | NR |
| SES/Education | ≥College-level education, n (%)  Empowered care: 166 (21), Team-based empowered care: 169 (21); C: 150 (19) |
| Type/Duration of Diabetes | Type: T2DM  Duration (years): 16.4 (9.8) |
| Diabetes Treatment | Medication, n (%): I: Empowered care, Team-based empowered care; C  RAAS inhibitors: I: 551 (69), 564 (71); C: 550 (69)  BP-lowering drugs: I: 659 (82), 676 (85); C: 668 (84)  Lipid-lowering drugs: I: 614 (77), 611 (77); C: 626 (79)  Noninsulin glucose-lowering drugs: I: 650 (81), 659 (83); C: 668 (84)  Insulin: I: 387 (48), 401 (50); C: 347 (44) |
| Comorbidities | Comorbidity, n (%): I: Empowered care, Team-based empowered care; C  General obesity: I: 495 (62), 477 (60); C: 495 (62)  Hypertension: I: 755 (94), 750 (94); C: 744 (94)  Dyslipidemia: I: 770 (96), 755 (95); C: 762 (96) |
| Outcome(s) Description | Primary: n (%) who attained multiple (at least 3 of 5) treatment targets: a hemoglobin A1c (HbA1c) level less than 7.0%, BP less than 130/80 mm Hg, a low-density lipoprotein (LDL) cholesterol level less than 1.8 mmol/L, a triglyceride level less than 1.7 mmol/L, and persistent use of RAAS inhibitors  Secondary: composite of incident cardiovascular, kidney, and cancer events in patients who did or did not attain multiple treatment targets.  self-monitoring of blood glucose at least once per week, regular exercise at least 3 times per week, and/or adherence to a balanced diet (yes or no) in the past 3 months |
| Type of Analysis | Intention-to-treat analysis: 2 or fewer nurse contacts at baseline and month 12 for the usual care group, additional 3 telephone contacts by nurses over 12 months for the empowered care group, and 6 or more clinic visits and/or nurse telephone contacts by the same physician-and-nurse team over 12 months for the team-based empowered care group; Per-protocol analyses: patients who fulfilled all inclusion and exclusion criteria, adhered to prespecified study procedures, and returned for reassessment at month 12; χ2 test; Fisher exact test; unpaired, 2-tailed t test; and analysis of variance for between-group comparisons; McNemar test for within-group comparisons. Poisson regression model was used to derive the risk ratios (RRs) and 95% CIs for the attainment of multiple treatment targets in the team-based empowered care group vs the usual care and empowered care groups, which were adjusted for site (model 1) and for site and baseline insulin use (model 2) attributed to between-group differences |
| Tools for Outcome Assessment | NR |
| Frequency of Outcome Assessment | Baseline, 12 months |
| Outcome Results | clinical, behavioral |
| Study Findings | This trial found that technology-assisted team-based care for 12 months improved the attainment of multiple treatment targets as well as empowerment in patients with DKD. |
| Chadosh Framework Category | Medical |

| Authors, Year | Poonprapai et al, 2022 |
| --- | --- |
| Purpose/Objective | To evaluate the effectiveness of family support-based intervention via a mobile application by pharmacists on clinical outcomes, family behaviour, diabetes knowledge, self-management practices and medication adherence in older adults with type 2 diabetes. |
| Study Design | Randomised controlled trial |
| Country/Setting | Thailand/hospital |
| Study Duration | 9 months |
| Inclusion/Exclusion Criteria | Inclusion: ≥65 years of age; diagnosis of type 2 diabetes; inadequate glycaemic control (glycosylated haemoglobin (HbA1c) level>7% or 53 mmol/mol); receipt of oral antihyperglycaemic agents; and having a family member as a caregiver. The eligible family members were ≥18 years old; a spouse, child or relative of the patient; and able to participate in this study by using mobile phone.  Exclusion: received insulin therapy |
| Sample Size | O: 166; I: 83; C: 83 |
| Intervention Description/Delivery | Description: received family-based intervention via a mobile app. The educational intervention was delivered via mobile app by pharmacists to family members. Afterwards, the family members would inform the patients of the contents received. The goal of the intervention was to facilitate family members’ assistance in the patients’ self-management tasks to achieve the diabetes goals.  Delivery: hybrid: mobile app, in-person |
| Control Description | Usual care |
| Follow Up (%) | I: 94; C: 95 |
| Mean Age (SD) | I: 67.36±5.72; C: 67.80±6.18 |
| Male (%) | I: 40; C: 41 |
| Race (%) | NR |
| SES/Education | Education, n (%): I; C  ≤Primary school: 63 (81); 59 (75)  Secondary school: 10 (13); 13 (17)  >Secondary school: 5 (6); 7 (9) |
| Type/Duration of Diabetes | Type: T2DM  Duration: I: 6.97±3.32; C: 6.87±3.20 |
| Diabetes Treatment | Antidiabetic medication, n (%): I; C  Metformin: 16 (21); 22 (28)  Glipizide: 5 (6); 3 (4)  Metformin+glipizide: 37 (47); 43 (54)  Glipizide+pioglitazone: 1 (1); 0  Metformin+glipizide+pioglitazone: 19 (24); 11 (14) |
| Comorbidities | Comorbidity, n (%): I; C  Hypertension: 73 (94); 71 (90)  Dyslipidaemia: 67 (86); 63 (80) |
| Outcome(s) Description | Primary: HbA1c  Secondary: family behaviour, both supportive and obstructive (such as nagging or arguing), diabetes knowledge, self-management practices, medication adherence, blood pressure |
| Type of Analysis | chi-square test for categorical variables and independent samples t-test for continuous variables, within-group diferences were tested using paired t-test, medication adherence scores between groups at diferent times were evaluated using split-plot ANOVA (or repeated measures ANOVA). |
| Tools for Outcome Assessment | Family behaviour: revised version of the Diabetes Family Behaviour Checklist  Diabetes knowledge: General Knowledge of Patients with Diabetes  Self-management practices: modifed version of the Summary of Diabetes SelfCare Activities Scale  Medication adherence: pill count formula: [(number of pills received—number of pills remaining)/number of  pills received]×100 |
| Frequency of Outcome Assessment | Baseline and at 9 months after the intervention. Adherence to medications was assessed at every visit, 3 months apart, until the end of the study. |
| Outcome Results | Clinical, knowledge, behavioural |
| Study Findings | Family support intervention via a mobile application by pharmacists is beneficial to diabetes care for older adults. |
| Chadosh Framework Category | Tailoring |

| Authors, Year | Vasconcelos et al, 2021 |
| --- | --- |
| Purpose/Objective | To evaluate the impact of adding food education sessions to an exercise programme on cardiovascular risk factors in middle-aged and older patients with type 2 diabetes (T2D) |
| Study Design | Randomised parallel-group trial |
| Country/Setting | Portugal/community-based |
| Study Duration | 9 months |
| Inclusion/Exclusion Criteria | Inclusion: T2D diagnosed at least for 6 months; aged between 50 and 80 years old; non-smokers; not engaged in supervised exercise; independent living in the community; medical recommendation for lifestyle intervention; known medical history; diabetes comorbidities under control (diabetic foot, retinopathy and nephropathy); no cardiovascular, respiratory and musculoskeletal contraindications to exercise; without major changes in gait and balance; not started insulin therapy in the past 3 months.  Exclusion: NR |
| Sample Size | O: 42; I: 23; C: 19 |
| Intervention Description/Delivery | Description: Diabetes em Movimento exercise programme plus concomitant food education sessions during 16 weeks based on American Diabetes Association (ADA) recommendations for dietary intake, and International Diabetes Federation (IDF) nutrition teaching modules. On each week, a different nutrition-related content was addressed by an exercise professional with professional qualification in the area of Nutrition through two sessions: a theoretical session of 15 min performed through an interactive teaching method before one exercise session (instructing participants in a way they are actively involved with their learning process); and dual-task strategies integrated in another exercise session (during aerobic exercise (brisk walking), patients had to interpret food labels or to give individual or group answers to nutritional questions through a traffic light system or multiple choice answer).  Delivery: in-person |
| Control Description | ‘Diabetes em Movimento’, a community-based exercise programme for patients with T2D, implemented during 9 months and consisted of three exercise sessions per week, 75min per session. Each session was performed at the municipal sports complex and organised according to the following structure: warm-up, aerobic exercise, resistance exercise, agility/balance exercise and flexibility exercise. |
| Follow Up (%) | I: 78; C: 79 |
| Mean Age (SD) | O: 65.4±5.9; I: 67.61 ± 5.37; C: 62.80 ± 5.52 |
| Male (%) | I: 50; C: 67 |
| Race (%) | NR |
| SES/Education | Educational level, n (%): I; C  ≤4 years of school: 11 (61); 9 (60)  5–9 years of school: 5 (28); 3 (20)  >9 years of school: 2 (11); 3 (20)  Personal net monthly income, n (%): I; C  <1000 €: 10 (55⋅6); 2 (13⋅3)  1000–2000 €: 6 (33); 7 (47)  >2000 €: 2 (11); 6 (40) |
| Type/Duration of Diabetes | Type: T2DM  Duration: I: 5.72 ± 4.63; C: 8.00 ± 5.72 |
| Diabetes Treatment | Medication, n (%): I; C  No medication: 1 (6); 0 (0)  Oral antidiabetics: 15 (83); 13 (87)  Oral antidiabetics + Insulin: 1 (6); 2 (13)  Insulin: 1 (6); 0 (0⋅0) |
| Comorbidities | Hypertension, n (%): I; C  Hypertension: 18 (100); 15 (100) |
| Outcome(s) Description | Glycaemic control: HbA1c; BMI; WC; FM; Blood pressure |
| Type of Analysis | χ2 test HbA1c, BMI and WC within groups.  analysis of variance (ANOVA) with repeated measures |
| Tools for Outcome Assessment | Glycaemic control: fasting (minimum of 8 h) venous blood analysis according to standard international laboratory methods  BMI: digital weight scale (SECA 778, SECA Corporation, Hamburg, Germany), stadiometer (SECA 220, SECA  Corporation, Hamburg, Germany)  WC: tape at the umbilical reference (SECA 201, SECA Corporation, Hamburg, Germany)  FM: bioelectrical impedance analysis (Tanita, BC-418 MA)  Blood pressure: automatic digital blood pressure device (BP-8800, Colin Corporation, Komaki, Japan) |
| Frequency of Outcome Assessment | Baseline, 9 months |
| Outcome Results | Clinical |
| Study Findings | The addition of a simple food education dietary intervention to an exercise programme improved body weight and composition, but not glycaemic control and blood pressure in middle-aged and older patients with T2D. |
| Chadosh Framework Category | Group |

| Authors, Year | Pai et al, 2021 |
| --- | --- |
| Purpose/Objective | To explore the effects of a health technology education program on long-term glycemic control and the self-management ability of adults with type 2 diabetes (T2D). |
| Study Design | Randomized controlled trial |
| Country/Setting | Taiwan/department of family medicine of a teaching hospital |
| Study Duration | 6 months |
| Inclusion/Exclusion Criteria | Inclusion: aged >/= 20 years who were on oral hypoglycemic medications (metformin ± sulfonylureas ± dipeptidyl peptidase-4 inhibitors ± a-glucosidase inhibitor) and were receiving routine diabetes shared care  Exclusion: other major diseases, such as myocardial infarction and stroke; undergoing hemodialysis; and severe physical disabilities |
| Sample Size | O: 108; I: 53; C: 55 |
| Intervention Description/Delivery | Description: Received routine shared care plus the health technology education program. Informative self-learning digital videos of health education were then provided to each subject in the experimental group, according to their individual learning needs, for self-study at home. The time needed for health education of each subject ranged from 20 to 40 min. In addition, each patient was given a wearable pedometer, instructions on how to use it, and the interactive diet-calculating software, so that they could calculate their weekly number of walking steps and daily consumption of calories and various nutrients. The experimental group also received monthly 10-minute phone consultations to answer any questions and discuss health-related needs.  Delivery: hybrid: virtual and phone |
| Control Description | Routine shared care |
| Follow Up (%) | I: 96; C: 96 |
| Mean Age (SD) | I: 66.34 ± 12.65; C: 66.16 ± 11.81 |
| Male (%) | I: 47; C: 47 |
| Race (%) | NR |
| SES/Education | Education: n (%): I; C  Illiterate: 10 (19); 10 (18)  Literate: 43 (81); 45 (82) |
| Type/Duration of Diabetes | Type: T2DM  Duration (years from disease diagnosis): n (%): I; C </=1 year: 9 (17); 4 (7) 1–5 years: 32 (60); 29(52) >/=5 years: 12(23); 22(41) |
| Diabetes Treatment | Medication use, n (%): I; C  Regular: 47 (89); 49 (89)  Irregular: 6 (11); 6 (11)  Diabetes drugs, n (%): I; C  1: 12(23); 17(31)  2: 37(69); 30(54)  >/=3: 4(8); 8(15) |
| Comorbidities | Comorbidity, Yes/No, n (%): I; C  Yes: 48 (90); 49 (89)  No: 5 (10); 6 (11) |
| Outcome(s) Description | Primary: HbA1c  Secondary: self-management ability |
| Type of Analysis | F-test and repeated measures analysis of variance |
| Tools for Outcome Assessment | HbA1c: Bio-Rad D-10TM Hemoglobin Analyzer (Bio-Rad Laboratories, Inc., CA, USA)  Self-management ability: Chinese version of Perceived Diabetes Self-Management Scale (PDSMS) |
| Frequency of Outcome Assessment | Baseline, 3 and 6 months |
| Outcome Results | Clinical, educational |
| Study Findings | The health technology education program was more effective than routine shared care alone in lowering HbA1c and increasing self-management ability in T2D patients. |
| Chadosh Framework Category | Feedback |

| Authors, Year | Chen et al, 2021 |
| --- | --- |
| Purpose/Objective | To explore the effects and feasibility of the peer-led self-management (PLSM) program for older adults with diabetes. |
| Study Design | Randomized controlled trial |
| Country/Setting | Taiwan/10 communities |
| Study Duration | 4 weeks |
| Inclusion/Exclusion Criteria | Inclusion: 65 years or older; diagnosis of T2DM; elementary or higher level of education, with basic literacy skills; and communicable in Mandarin or Taiwanese  Exclusion: Moderate or severe cognitive deterioration and diabetic retinopathy |
| Sample Size | O: 28; I: 14; C: 14 |
| Intervention Description/Delivery | Description: The peer-led self-management (PLSM) program, a theory-based intervention protocol, utilized Kolb’s experiential learning theory as the framework and Bandura’s self-regulation theory as the strategy. 90 min per week, over four consecutive weeks, group activity. In the experimental group, the participants attended the PLSM intervention program, received a self-management manual, and were instructed to continue the usual routine clinical care. Participants underwent a 4-week self-management program that required completion of tasks from the self-management manual, such as self-monitoring of diet and exercise.  Delivery: in-person |
| Control Description | Self-management manual and usual routine clinical care |
| Follow Up (%) | I: 100; C: 100 |
| Mean Age (SD) | O: 72.25 (4.62); I: 71.71 (3.83); C: 72.79 (5.38) |
| Male (%) | I: 14; C: 29 |
| Race (%) | NR |
| SES/Education | Education, n (%): I; C  Elementary: 9(64); 7 (50)  Middle school: 0; 0  High school: 3 (21); 3 (21)  Junior college: 2 (14); 1 (7)  University: 0; 3 (21) |
| Type/Duration of Diabetes | Type: T2DM  Duration (years): I: 13.21 (8.96); C: 12.43 (9.89) |
| Diabetes Treatment | NR |
| Comorbidities | NR |
| Outcome(s) Description | Primary: HbA1c, self-management, and self-efficacy  Secondary: total cholesterol, triglycerides, low density lipoprotein, high density lipoprotein, body weight, body mass index, and blood pressure |
| Type of Analysis | Inferential statistics used nonparametric statistics (Chi-square test, Fisher’s exact test, Wilcoxon signed-rank test, Mann-Whitney U test, and Friedman test) to compare the basic profiles of the two groups before and after and within groups. The Friedman test was used to assess within-group differences for self-efficacy, self-management, and physiological measures (including systolic pressures, diastolic pressures, body weight, and body mass index) collected at three time points before and after program intervention. Those with statistically significant differences were then further analyzed and compared using Bonferroni's method, at two time points. As for physiological measures (total cholesterol, high-density lipoprotein, triglycerides, low-density lipoprotein, and HbA1c) collected at two time points, the Wilcoxon signed-rank test was used for within-group comparison before and after study intervention. |
| Tools for Outcome Assessment | Self-efficacy: Chinese Version of the Diabetes Management Self-Efficacy Scale  Self-management: Diabetes Self-Management Instrument Short Form  HbA1c, total cholesterol, triglycerides, low-density lipoprotein, and high-density lipoprotein: collected  after 8 h of fasting and were tested by a professional laboratory certified by the Taiwan Accreditation Foundation  Blood pressure: sphygmomanometers  Body mass index: calibrated instruments |
| Frequency of Outcome Assessment | Baseline, after 4 weeks, 12 weeks after completion of the program intervention |
| Outcome Results | Clinical, knowledge, behavioral |
| Study Findings | This study suggests the feasibility of the program and affirmed the role of peer leaders in improving self-management of peer participants over 65 years of age. |
| Chadosh Framework Category | Feedback |

| Authors, Year | Borba et al, 2020 |
| --- | --- |
| Purpose/Objective | To assess the effects of a problematization educational intervention to promote healthy habits in elderly people with diabetes |
| Study Design | Randomized clinical trial |
| Country/Setting | Brazil/Family Health Units (n=8) |
| Study Duration | 6 months |
| Inclusion/Exclusion Criteria | Inclusion: Elderly people diagnosed with DM2 and assisted by such Family Health Units  Exclusion: elderly people with DM, institutionalized or wheelchair users, with impaired communication and/or cognition, chronic complications of DM in advanced stages recorded in the health record and mobility difficulties |
| Sample Size | O: 202; I: 101; C: 101 |
| Intervention Description/Delivery | Description: The educational intervention was based on problematization pedagogical methodology. Six educational  meetings, with an average of 12 to 15 participants, with monthly frequency and an average duration of two hours. To organize the educational activities, a teaching plan was developed for the central themes and group work strategies: understanding DM and its complications (educational video, educational game Simulation Roulette, problem situation/stage play); choice of healthy foods within the food groups (Food Pyramid poster, figure of regional foods and fruits, household measures, problem situation/theatrical staging, educational game Traffic Light Labelling); physical activity and self-care with food and feet (colorful balloons, music, activities with movement, dialogues, posters, educational game “Right” or “Wrong” to promote self-care with food and feet, making the Daily Watch); diet, light, zero-calory food and labels (educational video, figures of food labels, dialogued exhibition, problem situation/theatrical staging); consumption of healthy foods (verbal expressions of significant experiences, food figures, problem situation/theatrical staging with the context of a self-service for healthy dish assembly); preparation and preservation of food (glitter balloons, newspaper clippings, dialogue exhibition, figures, problem situation/theatrical staging).  Delivery: hybrid: in-person, virtual |
| Control Description | Conventional care |
| Follow Up (%) | I: 53; C: 47 |
| Mean Age (SD) | I: 66.0; C: 66.0 |
| Male (%) | I: 33; 21 |
| Race (%) | NR |
| SES/Education | Education, (%): I; C  Education (≤ 8 years): 77; 83 |
| Type/Duration of Diabetes | Type: T2DM  Duration (months): I: 108.0; C: 108.0 |
| Diabetes Treatment | NR |
| Comorbidities | NR |
| Outcome(s) Description | Knowledge about DM; diabetes attitudes; food consumption; physical activity; smoking; alcoholic beverage consumption; weight; BMI; WC; A1C |
| Type of Analysis | Pearson’s chi-square test, Kolmogorov-Smirnoff test, Student’s t-test, or Mann-Whitney U test  Assessment of the effect of time on each variable was performed using Generalized Linear Models (GLM), with the Bonferroni test for multiple comparisons. Pearson’s chi-square test and Fisher’s exact test were used to analyze comparisons of proportions. |
| Tools for Outcome Assessment | Knowledge about DM: Diabetes Knowledge Scale (DKN-A)  Diabetes Attitudes: Diabetes Attitudes Questionnaire (ATT-19)  Food consumption: two 24-hour recalls, The Healthy Eating Index adapted for the elderly  Physical activity: International Physical Activity Questionnaire (IPAQ)  Smoking: cigarette use  Alcoholic beverage consumption: alcohol abuse frequency  Weight: digital electronic scale, Tanita  WC: inelastic measuring tape  Glycemic control: A1C |
| Frequency of Outcome Assessment | Baseline, 3 months, 6 months |
| Outcome Results | Educational, knowledge, clinical, behavioral |
| Study Findings | The problematic group intervention is a potential strategy to promote physical activity, less consumption of oils and fats, greater variety of diet, increased knowledge about DM and positive attitude towards self-care. |
| Chadosh Framework Category | Group |

| Authors, Year | Yang et al, 2020 |
| --- | --- |
| Purpose/Objective | To evaluate the effectiveness of a group visit intervention in comparison with the usual care for elderly patients with type 2 diabetes in a community. |
| Study Design | Randomized quasi-experimental |
| Country/Setting | China/community-based health center |
| Study Duration | 6 months |
| Inclusion/Exclusion Criteria | Inclusion: Men and women aged 60 years or older with type 2 diabetes confirmed by medical records; sufficient Chinese language skills; and written informed consent.  Exclusion: Severe diabetes-related complications, determined using diagnostic criteria for proliferative retinopathy, were at the IV stage of nephropathy or had creatinine levels above 2 mg/dl, had a diabetic foot above grade 1, or had dementia that indicated serious cognitive impairment. |
| Sample Size | O: 109; I: 55; C: 54 |
| Intervention Description/Delivery | Description: The group visits were modeled after the Cooperative Health Care Clinics (CHCC) developed by Beck et al. This group visit model involves an interdisciplinary health care team to incorporate expertise in a variety of fields. The team consisted of one diabetes nurse, one primary care physician, one dietitian, and one psychologist, who were all involved in the intervention. Team members alternated leading the group education sessions based on their areas of expertise. In addition, they assisted patients in developing and reviewing individual action plans regarding drugs, diet, and emotion management. The team also asked each participant to share their progress toward their individual goals since the last visit. Each group visit session lasted approximately 2 h, and included a 15 min warm-up and feedback period, a 40-minute presentation of diabetes-related education, a brief 10-minute break, and a 15-minute interaction period, followed by 40 min of one-on-one consultations with the interdisciplinary expertise as needed. Only one or two core topics are  covered in each group visit, with a direct connection to the individualized action plan of the group visits.  Delivery: in-person |
| Control Description | Some form of diabetes self-management education provided by the diabetes nurse educator as usual. The education consisted of five monthly lectures on nutrition, exercise, medications, and complications of diabetes, along with a telephone follow-up. Brochures about disease knowledge were also distributed. |
| Follow Up (%) | I: 80; C: 80 |
| Mean Age (SD) | O: 69.72 (5.24); I: 70.55 (5.17); C: 68.89 (5.23) |
| Male (%) | I: 33; C: 46 |
| Race (%) | NR |
| SES/Education | Education, n (%): I; C  Middle school or less: 30 (55); 24 (44)  High school: 18 (33); 13 (24)  College graduate or higher: 7(13); 17(32)  Household monthly income, n (%): I; C  <1000: 4 (7); 3 (6)  1000-3000: 26 (47); 22 (41)  3000-6000: 22 (40); 21 (39)  >6000: 3 (5); 8 (15) |
| Type/Duration of Diabetes | Type: T2DM  Duration: years n (%): I; C <10: 35 (64); 29 (54) 10-20: 13 (24); 21 (39) ≥20: 7 (13); 4 (7) |
| Diabetes Treatment | NR |
| Comorbidities | NR |
| Outcome(s) Description | Primary: HbA1C  Secondary: diabetes-related knowledge, self-management behaviors, and self-efficacy |
| Type of Analysis | Two-sample T-test, and Pearson chi square test for categorical variables. Repeated measures analysis of variance was used to  assess intervention efficacy. Missing data, assumed to be missing at random, were statistically imputed by Expectation Maximization (EM) using SPSS’ missing value analysis. |
| Tools for Outcome Assessment | HbA1C: blood sample was collected for HbA1C testing at the health care center in the community  Diabetes-related knowledge: modified version of the Diabetes Knowledge Questionnaire (DKQ)  Self-management behaviors: modified version of the type 2 Diabetes Self-Care Scale (2-DSCS)  Self-efficacy: Chinese version of the 6- item Self-Efficacy for Managing Chronic Disease Scale |
| Frequency of Outcome Assessment | Baseline, 3 months, 6 months |
| Outcome Results | Knowledge, clinical, behavioral |
| Study Findings | The group visits model increased diabetes knowledge and self-efficacy and improved patients’ self-management behavior. The model was found suitable for helping these elderly patients with type 2 diabetes achieve effective self-management. |
| Chadosh Framework Category | Group |

| Authors, Year | Sun et al, 2019 |
| --- | --- |
| Purpose/Objective | To investigate the use of mobile phone-based telemedicine apps for management of older Chinese patients with type 2 diabetes mellitus (T2DM). |
| Study Design | Randomized Controlled Trial |
| Country/Setting | China/outpatient endocrinology department of the First Affiliated Hospital of Jilin University |
| Study Duration | 6 months |
| Inclusion/Exclusion Criteria | Inclusion: Older than 65 years, glycated hemoglobin (HbA1c) level 7.0% to 10.0%, and the ability to use a mobile phone  Exclusion: Illiteracy, abnormal liver and kidney function, severe diabetic complications, use of insulin pumps, and participation in other clinical trials |
| Sample Size | O: 91; I: 44; C: 47 |
| Intervention Description/Delivery | Description: Patients in the intervention group were provided training to independently use the mHealth management app and upload the glucometer data, which was then automatically transmitted to the medical server. The medical teams logged on to the system and sent medical advice and reminders to patients to monitor their glucose levels via the personal messaging app or telephonically every 2 weeks.  Patients in the intervention group used the app-based diet management software to input daily dietary intake. The dietitian received the daily dietary record of each patient via the mHealth app. On the basis of the analysis of this information, once-monthly dietary recommendations were sent from the dietitian to patients in the intervention group.  each patient in the intervention group was provided with guidance related to aerobic and resistance-based exercises.  Delivery: hybrid: virtual, in-person |
| Control Description | Received a free glucometer and were followed up through conventional outpatient clinic appointments. The control group received dietary guidance from dietitians. guidance related to exercise was provided during face-to-face dietary counseling session during clinic visits. |
| Follow Up (%) | I: 100; C: 100 |
| Mean Age (SD) | median (IQR) – I: 67.9 (66-71); C: 68.04 (66-72) |
| Male (%) | I: 43; C: 38 |
| Race (%) | NR |
| SES/Education | NR |
| Type/Duration of Diabetes | Type: T2DM  Duration (years) I: 11.19 (6.39); C: 11.52 (7.73) |
| Diabetes Treatment | NR |
| Comorbidities | NR |
| Outcome(s) Description | Blood glucose; HbA1c; total cholesterol; triglyceride; HDL; LDL; BMI; blood pressure |
| Type of Analysis | Between-group differences to normally distributed variables were assessed using an independent sample t test, whereas those to nonnormally distributed variables were assessed using a Mann-Whitney U test. For intragroup comparison, normally distributed variables were tested by paired ttest and nonnormally distributed variables were tested by Wilcoxon rank-sum test. |
| Tools for Outcome Assessment | NR |
| Frequency of Outcome Assessment | Baseline, 3 months, 6 months |
| Outcome Results | Clinical |
| Study Findings | Mobile phone–based telemedicine apps help improve glycemic control in older Chinese patients with T2DM. |
| Chadosh Framework Category | Feedback |

| Authors, Year | De Greef et al, 2011 |
| --- | --- |
| Purpose/Objective | To investigate whether a 12-week pedometer-based PA intervention individually delivered by a trained GP during patient visits can be as effective as group delivery by a behavioral expert. |
| Study Design | Three-Arm Randomized Controlled Trial |
| Country/Setting | Belgium/Primary care (GP offices) |
| Study Duration | 12 weeks |
| Inclusion/Exclusion Criteria | Inclusion: (1) ≥ 6 months post diagnosis of type 2 diabetes (disease is stabilized); (2) age ≤80 years (if >80, too much risk for injuries); (3) BMI 25-35 kg/m² (obese patients with BMI >35 kg/m² need to be referred to other treatments); (4) pharmaceutically treated for type 2 diabetes; (5) HbA1c ≤12% (if >12%, need to be referred to more intensive treatment); (6) no documented physical or medical PA limitations; (7) Dutch speaking.  Exclusion: NR |
| Sample Size | O: 67; GP (individual): 22; CBT (group): 21; C: 24 |
| Intervention Description/Delivery | Description: GP-Delivered Intervention - Three individual 15-min face-to-face consultations. The content of this intervention was similar as this of the CBT group intervention. Since it was not possible for the GPs to quote all topics in the limited time they had for each patient (15 min vs. 90 min of group session), they were asked to focus on pedometer use, goal-setting, decisional balance, and relapse prevention.  Behavioural Expert-Delivered CBT Group - Three 90-min group counseling sessions over a 12-weeks period (one session every 3 weeks). The group sessions were based on cognitive-behavioral therapy [66], the Diabetes Prevention Program [67], the First Step Program [68], and motivational interviewing [69]. The group sessions started with a motivational interviewing phase. In the first session, each participant received a pedometer. The aim of this session was to increase knowledge about the benefits of PA. During this session, discussions with the participants about benefits and risks took place. Commitment to a lifestyle change plan was strengthened by learning time management skills. The participants together with the behavioral expert developed a lifestyle change plan in which "where, when, and how" of the planned behavior changes was explained. Patients were encouraged to take responsibility for their own goal-setting.  In a second session, the first experiences of the participants with the pedometers as a motivation tool and goal-setting were exchanged. Benefits and barriers of PA as well as strategies and tips on how to overcome the barriers were discussed. In the last session, the focus was on learning relapse prevention skills. The group format was highly interactive; ideas were introduced by the participants and discussed within the group. During the group sessions, the participants received personal goal-setting sheets and a decisional balance table.  Delivery: In-person |
| Control Description | The control group only received general care from their GP. |
| Follow Up (%) | GP (individual): 100; CBT (group): 95.2; C: 91.6 |
| Mean Age (SD) | O: 67.4 (9.3); GP (individual): 66.6 (9.5); CBT (group): 70.0 (6.3); C: 66.0 (11.1) |
| Male (%) | GP (individual): 77.3; CBT (group): 61.9; Control: 70.8 |
| Race (%) | NR |
| SES/Education | NR |
| Type/Duration of Diabetes | Type: T2DM  Duration: >5 years: Overall: 35.5%; GP (individual): 33.3%; CBT (group): 31.6%; Control: 40.9% |
| Diabetes Treatment | Insulin - GP (individual): 0%; CBT (group): 0%; Control: 4.5%, Oral Medication - GP (individual): 100%; CBT (group): 95%; Control: 77.3%, Combination - GP (individual): 0%; CBT (group): 5%; Control: 18.2% |
| Comorbidities | Reported health problems in Characteristics table (unspecified):  Health problems - Overall: 90.6%; GP (individual): 95.2%; CBT (group): 90.0%; Control: 87.0%  None - Overall: 9.4%; GP (individual): 4.8%; CBT (group): 10.0%; Control: 13.0% |
| Outcome(s) Description | Pedometer-Based PA: An activity log was used to record the steps taken and the type and duration of non-walking activities [62]. Participants were asked to complete the activity log at the end of each day. Following established guidelines, participants were instructed to add 150 steps to the daily total for every minute actively spent biking or swimming [62]. At the end of each measurement period, all participants gave their activity log (with an anonymous study number) to their GP.  Self-Reported PA: Self-reported PA was assessed with the Dutch interview version of the long International PA Questionnaire (IPAQ)(last 7 days). In the questionnaire, frequency (number of days) and duration (hours and minutes per day) of PA in different domains (work, transportation, leisure time, and housekeeping) were queried.  Health Measurements: Body weight, standing height, standing waist circumference, biochemical data (fasting plasma glucose, HbA1c, total cholesterol) |
| Type of Analysis | Quantitative |
| Tools for Outcome Assessment | Self-Reported PA: International Physical Activity Questionnaire  (IPAQ)  Body Weight: SECA 813 Robusta balance  Height: Harpenden stadiometer |
| Frequency of Outcome Assessment | Baseline and immediately following the 12-week intervention |
| Outcome Results | Clinical |
| Study Findings | Results demonstrated that the group counseling was more effective in increasing pedometer-determined PA compared to both the individual consultation and the control condition; Group counseling participants had a defined increase in steps/day (+1,706) compared to those of the other conditions; no improvements in health outcomes were observed for the patients assigned to the group counseling intervention. |
| Chadosh Framework Category | Psychological Emphasis |

| Authors, Year | Sharifirad et al, 2013 |
| --- | --- |
| Purpose/Objective | To evaluate the effects of a nutritional education program on cardiovascular risk among elderly patients with type 2 diabetes (T2D). |
| Study Design | Randomized controlled trial |
| Country/Setting | Iran/Diabetes clinics |
| Study Duration | 12 weeks |
| Inclusion/Exclusion Criteria | Inclusion: Type 2 diabetes for at least 1 year, age >=60 years, and the absence of any cognitive and/or motor disabilities  Exclusion: NR |
| Sample Size | O: 100; I: 48; C: 49 |
| Intervention Description/Delivery | Description: Four 70-min. sessions (1 month duration): The BASNEF model (which includes beliefs, attitudes, subjective norms and enabling factors) was used to design the educational intervention in three parts: (i) changing nutritional attitudes by increasing knowledge and improving nutritional habits; (ii) taking subjective norms into consideration by providing group sessions with the patient`s family to discuss dietary intake, an educational session with staff (physician, endocrinologist, diabetes nurse, and nutritionist) familiar with methods that can be used to change nutritional attitudes among elderly T2D patients, and telephone follow-up 4 to 6 weeks after the educational intervention to respond to any questions and to re-emphasize the education material; and (iii) emphasizing enabling factors. Nutritional education was provided by the nutritionist. The goals of the sessions were to increase the frequency of meals and to decrease the amount of fat and simple sugars consumed while increasing fruit and vegetable intake in the intervention group. The final session was conducted with the patient in the presence of his/her family and the research staff. Information was provided to the patients via lectures and subsequent discussions. Education pamphlets were given to the patient's family at the end of education session to involve them in the intervention.  Delivery: In-person, telephone follow-up |
| Control Description | The usual care was continued for all patients, including periodic visits, free biochemical tests, and recording their medication use. |
| Follow Up (%) | I: 96; C: 98 |
| Mean Age (SD) | I: 67.3 (3.5); C: 66.8 (3.6) |
| Male (%) | I: 33.3; C: 36.7 |
| Race (%) | NR |
| SES/Education | >Primary School - I: 11 (22.9); C: 14 (28.6), ≤Primary School - I: 37 (77.1); C: 35 (71.4) |
| Type/Duration of Diabetes | Type: T2DM  Duration: I: 15 (2) years; C: 13 (2) years |
| Diabetes Treatment | NR |
| Comorbidities | NR |
| Outcome(s) Description | Lipid profiles (HDL, LDL, TG), Weight, Height, WHR, BMI, SBP, DBP |
| Type of Analysis | Quantitative |
| Tools for Outcome Assessment | Blood pressure: Sphygamomanometer  Weight: SECA 760 (SECA, Hamburg, Germany)  Height, WHR: Non-stretchable measuring tape  Lipid profiles: Commercially available kits (Amylase Kit 2010; Pars Azmun, Tehran, Iran) and an autoanalyzer (BT3000; Biotechnical, Rome, Italy) |
| Frequency of Outcome Assessment | Baseline and immediate post-intervention |
| Outcome Results | Clinical |
| Study Findings | Short-term nutritional education based on the BASNEF educational model improves serum triglyceride levels and anthropometric indices in elderly patients with T2D. |
| Chadosh Framework Category | Psychological emphasis |

| Authors, Year | Munshi et al, 2013 |
| --- | --- |
| Purpose/Objective | To evaluate whether assessment of barriers to self-care and strategies to cope with these barriers in older adults with diabetes is superior to usual care with attention control. |
| Study Design | Randomized controlled trial |
| Country/Setting | USA/ The Joslin Diabetes Center and the Beth Israel Deaconess Medical Center |
| Study Duration | 1 year |
| Inclusion/Exclusion Criteria | Inclusion: Patients aged 69 years with type 1 or type 2 diabetes of at least 1 year duration with poorly controlled diabetes (A1C >8%).  Exclusion: terminal diseases, living >25 miles from Boston, living in an institutional setting (e.g., nursing home, group home), and inability to complete outcome assessments (e.g. poor vision, severe cognitive decline, unable to speak, read, or write English). |
| Sample Size | O: 100; I: 70; C: 30 |
| Intervention Description/Delivery | Description: The patients in the intervention group underwent evaluation for barriers to self-care by a diabetes educator well versed with age-specific barriers; a geriatric diabetes team (GDT), consisting of a geriatric diabetologist, a diabetes educator, and a nutritionist, identified strategies to help patients cope with their barriers after consideration of patients' clinical and psychosocial environments and comorbid conditions. The strategies were designed to optimize patients’ ability to perform self-care leading to better adherence with treatment recommendations given by their medical providers. The strategies to cope with barriers were provided to the patients in intervention group via two methods (further randomization).  Intervention 1: An office-based diabetes educator provided the strategy by phone calls, speaking with patients up to 11 times during intervention period. The initial phone call included educating patients regarding their barriers and providing strategy options to cope with these barriers. Follow-up phone calls included continued assessment and encouragement to cope with barriers.  Intervention 2: A non-health professional care manager, trained by GDT, provided coping recommendations. The recommendations were conveyed to the patients in this group by the care manager. The care manager visited the patients’ homes to assess safety issues or other needs not known to the study team and helped the patients and caregivers with all aspects of care coordination, including making medical appointments and arranging transportation. Patients in this group received phone contact from the care manager as often as needed during the intervention period.  Delivery: In-person, telephone |
| Control Description | An educator (different from the one involved in the intervention) called participants in this group for a total of 11 times within the first 6 months to provide similar attention time; educator did not provide any diabetes-related advice or strategies and only discuss non-diabetes-related life events. |
| Follow Up (%) | I: 6-month - 97; 12-month: 96; C: 93 |
| Mean Age (SD) | O: 75 (5); I: 75 (5); C: 75 (5) |
| Male (%) | I: 43;% C: 53% |
| Race (%) | White - I: 76%; C: 80% |
| SES/Education | Education (years) - I: 15 (3); C: 14 (3) |
| Type/Duration of Diabetes | Type: T1DM & T2DM  Duration (years) - I: 20 (12); C: 23 (14) |
| Diabetes Treatment | Oral Agents - I: 10%; C: 7%; Insulin - I: 44%; C: 57%; Combination - I: 46%; C: 37% |
| Comorbidities | NR |
| Outcome(s) Description | Clinical measures (HbA1c, BP, BMI, Lipids, Compliance to diabetes-related behaviours, self-care behaviours, dietary assessment, cognition), Functional measures (AODL, # of falls, 6MWT, gait/balance), Psychosocial measures (geriatric depression, diabetes-related distress, social resource assessment), and Economic measures (# of ED visits, hospitalizations, outpatient care utilizations [clinic visits]) |
| Type of Analysis | Quantitative |
| Tools for Outcome Assessment | Clinical: Self-Care Inventory-R (SCI-R), Determine Your Nutritional Health Checklist, Clock-in-a-box test, Trail-Making Test A and B, Verbal fluency test  Functional: 6-minute walk test (6MWT), Tinetti test  Psychosocial: Geriatric Depression Scale (GDS), Problem Areas in Diabetes (PAID) test, Older Americans Resources and Services Multidimensional Functional Assessment Questionnaire |
| Frequency of Outcome Assessment | A1C: Baseline, 3 months, 6 months, 12 months  All Other Outcomes: Baseline, 6 months, and 12 months |
| Outcome Results | Clinical, educational, cognitive |
| Study Findings | The most common barrier was inadequate medications, primarily due to older patients' reluctance to make changes in insulin doses between clinic visits or during illnesses; contact with an educator improves HbA1C, self-care frequency, maintains functionality, and lowers distress in this population. |
| Chadosh Framework Category | Feedback |

| Authors, Year | Bond et al, 2007 |
| --- | --- |
| Purpose/Objective | To investigate the impact of a 6-month web-based intervention on the physical outcomes associated with diabetes management in older adults |
| Study Design | Randomized controlled trial |
| Country/Setting | USA/At home (Internet-based) |
| Study Duration | 6 months |
| Inclusion/Exclusion Criteria | Inclusion: age 60 years or older, having been diagnosed with diabetes (type 1 or type 2) for at least 1 year, living independently in the community, and oral fluency in English.  Exclusion: moderate or severe cognitive, visual, or physical impairment or the presence of severe comorbid disease (end-stage renal disease, blindness, terminal cancer) |
| Sample Size | O: 62; I: 31; C: 31 |
| Intervention Description/Delivery | The intervention consisted of a program designed to be delivered via the Internet to improve the participants diabetes self-management behaviors by using behavioral and motivational strategies and cues to modify perceptions of self-efficacy and personal beliefs regarding the  ability to affect the progress of the disease and change personal behavior. The patient’s role in maintaining health and the importance of setting goals and using problem solving skills to overcome barriers was emphasized. Additional strategies included instruction in disease  management, diet, and exercise, and the introduction of interventions to deal with the physical and emotional demands of the disease. The active intervention served as an adjunct to the usual care provided by each subject’s provider. The primary care physicians of subjects in both conditions retained full responsibility and control over their patients’ care.  The interaction between the study nurse and active invention participants occurred using both synchronous communication (instant messaging and chat) and asynchronous communication (e-mail and a bulletin board). In addition, participants accessed a study web site (www.diabetes-takecharge.org) to enter their blood sugar readings, exercise programs, weight changes, blood pressure, and medication data. The study nurse accessed participants’ logs to monitor changes in their self-management patterns. As part of the intervention, the study nurse contacted the participant via e-mail or through instant messenger and/or chat when there were changes in blood sugar patterns that needed problem-solving to resolve. The weekly online educational discussion group treatment component was delivered by the principal investigator through a weekly online or e-mail communication using MSN Messenger software provided by Microsoft Corp (Redmond, WA). The content for these weekly educational discussion sessions was developed by using resources available from the National Institutes of Health and the American Diabetes Association.  Delivery: Virtual |
| Control Description | Participants in the control group received their standard diabetes care from their provider. No educational or training materials associated with the intervention were provided to the control group. Participants in the control group had access to educational materials/classes provided by their health provider through traditional face-to-face classroom methods and/or via the Internet. |
| Follow Up (%) | NR |
| Mean Age (SD) | I: 66.2 (5.7); C: 68.2 (6.2) |
| Male (%) | I: 58; C: 52 |
| Race (%) | I: 87; C: 86 |
| SES/Education | Years of Education (mean) - I: 15.8 (1.5); C: 15.9 (2.2), Annual Income <$40,000 - I: 47%; C: 48%, Annual Income >$40,000 - I: 53%; C: 52% |
| Type/Duration of Diabetes | Type: T1DM & T2DM  Duration: Years (mean) - I: 16.1 (10.5); C: 17.8 (11.7) |
| Diabetes Treatment | NR |
| Comorbidities | NR |
| Outcome(s) Description | HbA1C; HDL; Total cholesterol; Weight; Systolic/diastolic blood pressure |
| Type of Analysis | Quantitative |
| Tools for Outcome Assessment | HbA1c: Single-use home HbA1c testing kit  BP: a blood pressure device with various-size cuffs  Weight: a calibrated scale (Tanita Corp., Arlington Heights, IL);  Total and HDL cholesterol: Cholestech (Hayward, CA) LDXÂ® analyzer  Self-Administered Comorbidity Questionnaire |
| Frequency of Outcome Assessment | Baseline and immediate post-intervention (6 months) |
| Outcome Results | Clinical |
| Study Findings | We found that participants who received a 6-month diabetes web-based intervention did improve on their HbA1c, systolic blood pressure, weight, HDL, and total cholesterol levels compared with the control group. In addition, regardless of HbA1c level at baseline (above or below 7.5%), active treatment participants showed improvements on their HbA1c. |
| Chadosh Framework Category | Psychological emphasis |

| Authors, Year | Lim et al, 2011 |
| --- | --- |
| Purpose/Objective | To determine if an individualized interactive u-healthcare service using the advanced information technology of the CDSS rule engine enabled more effective glucose control for an elderly population. |
| Study Design | Randomized controlled trial |
| Country/Setting | Korea/Outpatient clinic |
| Study Duration | 6 months |
| Inclusion/Exclusion Criteria | Inclusion: aged ≥60 years, diagnosed with type 2 diabetes for ≥ 1 year, A1C level was 6.5-10.5%  Exclusion: Patients with severe diabetes complications (e.g., diabetic foot or severe diabetic retinopathy); liver dysfunction (aspartate aminotransferase or alanine aminotransferase>2.5 times the reference level); or renal dysfunction (serum creatinine >132mmol/L [1.7 mg/dL]); or other medical problems that could affect study results or trial participation, patients without a text message function on their cellular phone or who were unable to use text messages for any reason |
| Sample Size | O: 154; U-healthcare: 51; SMBG: 51; C: 52 |
| Intervention Description/Delivery | Description: We provided pertinent diabetes education, including a therapeutic lifestyle change program, to standardize every patient’s education level and practice of diabetes management. A specialized diabetes management  team consisting of well-trained professionals, including diabetologists, nurses, dietitians, and exercise trainers, organized and directed patient education.  The SMBG group was advised to measure their blood glucose level at least 8 times a week ( ≥3 at fasting, ≥3 postprandial, and ≥2 bedtimes). The u-healthcare group was educated to use PSTN-connected glucometer to measure their blood glucose level at the same frequency as the SMBG group and to start short message service (SMS) on their mobile phone to receive messages from the CDSS rule engine server.  Delivery: Hybrid (in-person training, virtual [at home] intervention) |
| Control Description | After the pertinent diabetes education (given to all participants), individuals in the control group did not receive an intervention and were advised to follow-up according to their current medical care. |
| Follow Up (%) | U-healthcare : 96.1; SMBG: 92.2; C: 92.3 |
| Mean Age (SD) | U-healthcare: 67.2 (4.1); SMBG: 67.2 (4.4); C: 68.1 (5.5) |
| Male (%) | U-healthcare: 45; SMBG: 43; C: 37 |
| Race (%) | NR |
| SES/Education | Education level None n (%) - U-healthcare: 2 (3.9); SMBG: 3 (5.8); C: 1 (1.9), Primary School - U-healthcare: 10 (19.6); SMBG: 8 (15.4); C: 11 (21.2), Junior High School - U-healthcare: 20 (39.2); SMBG: 21 (40.4); C: 19 (36.5), ≥ High School - U-healthcare: 19 (37.3); SMBG: 20 (38.5); C: 21 (40.4) |
| Type/Duration of Diabetes | Type: T2DM  Duration: (years) U-healthcare: 14.1 (10.1); SMBG: 15.4 (8.3); C: 15.8 (10.7) |
| Diabetes Treatment | Medication for glucose control: Sulfonylurea n (%) - U-healthcare: 29 (58); SMBG: 24 (56); C: 28 (48), Metformin - U-healthcare: 34 (68); SMBG: 30 (65.2); C: 28 (56), Thiazolidinedione - U-healthcare: 4 (8); SMBG: 8 (16); C: 3 (6), Dipeptidyl peptidase - U-healthcare: 6 (12); SMBG: 11 (22); C: 6 (12), alpha-Glucosidase inhibitor - U-healthcare: 9 (18); SMBG: 13 (26); C: 12 (22.7), Insulin - U-healthcare: 12 (24); SMBG: 12 (24); C: 19 (38) |
| Comorbidities | NR |
| Outcome(s) Description | Primary Outcome: A1C  Secondary Outcomes: Frequency of SMBG, Episodes of hypoglycemia, Anthropometrics (Weight; BMI), Biochemical parameters (Fasting glucose; Postprandial glucose; Total cholesterol; Triglyceride; HDL cholesterol; LDL cholesterol) |
| Type of Analysis | Quantitative |
| Tools for Outcome Assessment | Clinical decision support system; glucometers (GlucoDr Supersensor,  AGM-2200, Allmedicus, Korea) |
| Frequency of Outcome Assessment | Baseline, 3 months, and 6 months (immediate post-intervention) |
| Outcome Results | Educational, clinical |
| Study Findings | The CDSS-based u-healthcare service achieved better glycemic control with less hypoglycemia than SMBG and routine care and may provide effective and safe diabetes management in the elderly diabetic patients. |
| Chadosh Framework Category | Tailoring |

| Authors, Year | Weinstock et al, 2011 |
| --- | --- |
| Purpose/Objective | The Informatics for Diabetes Education and Telemedicine (IDEATel) project randomized ethnically diverse underserved older adults with diabetes to a telemedicine intervention or usual care. Intervention participants had lower A1C levels over 5 years. New analyses were performed to help better understand this difference. |
| Study Design | Randomized controlled trial |
| Country/Setting | USA/ Primary care provider practices, at home |
| Study Duration | 5 years |
| Inclusion/Exclusion Criteria | Inclusion: Medicare beneficiaries with diabetes living in federally designated Medically Underserved or Health Professional Shortage Areas, fluent in English or Spanish, who provided informed consent.  Exclusion: Included moderate or severe cognitive impairment and severe comorbid conditions |
| Sample Size | O: 1665; I: 844; C: 821 |
| Intervention Description/Delivery | Description: Telemedicine subjects received a home telemedicine unit to videoconference with a diabetes educator every 4–6 weeks for self-management education, review of transmitted home blood glucose and blood pressure measurements, individualized goal-setting, and access to educational web pages created by the American Diabetes Association in English and Spanish.  Delivery: Virtual |
| Control Description | Usual care |
| Follow Up (%) | NR |
| Mean Age (SD) | O: Age at randomization – 71 |
| Male (%) | O: 37 |
| Race (%) | O (White): 50 |
| SES/Education | Education (years) in overall sample categorized by race - White: 11.99 (2.86); Black: 10.49 (2.93); Hispanic: 6.33 (3.74) |
| Type/Duration of Diabetes | Type: NR  Duration: Overall (mean): 11 years; <5 years (%) - White: 32.28; Black: 25.40; Hispanic: 39.74, 5-9 years (%) - White: 20.58; Black: 21.77; Hispanic: 18.97, 10-14 years (%) - White: 15.96; Black: 13.71; Hispanic: 19.49, ≥ 15 years (%) - White: 29.72; Black: 37.50; Hispanic: 31.28 |
| Diabetes Treatment | Oral agents alone (%) - White: 36.66; Black: 39.52; Hispanic: 36.24, Oral combination therapy (%) - White: 30.69; Black: 24.60; Hispanic: 30.77, Insulin alone (%) - White: 11.08; Black: 18.15; Hispanic: 9.91, Insulin and pills (%) - White: 14.49; Black: 10.89; Hispanic: 16.07, Diet alone (%) - White: 7.06; Black: 6.45; Hispanic: 6.84 |
| Comorbidities | NR |
| Outcome(s) Description | Primary Outcome: HbA1C  Secondary: BMI, urine microalbumin-to-creatinine ratio, depression, comorbidities, social engagement, QoL |
| Type of Analysis | Quantitative |
| Tools for Outcome Assessment | Depression: SHORT-Comprehensive Assessment and Referral Evaluation depression scale  Comorbidities: Charlson Comorbidity Index  Social Engagement: Lubben Social Network scale  QoL: General Health Short Form (SF-12)  A1C: Boronate Affinity Chromatography (Primus CLC 385). |
| Frequency of Outcome Assessment | Annually |
| Outcome Results | Clinical |
| Study Findings | Racial/ethnic disparities were observed in this cohort of underserved older adults with diabetes. The IDEATel telemedicine intervention was associated with improvement in glycemic control, particularly in Hispanics, who had the highest baseline A1C levels, suggesting that telemedicine has the potential to help reduce disparities in diabetes management. |
| Chadosh Framework Category | Tailoring |

| Authors, Year | Braun et al, 2009 |
| --- | --- |
| Purpose/Objective | To evaluate the effectiveness of a new structured diabetes teaching and treatment programme (DTTP) with specific didactical approaches and topics for geriatric patients with diabetes mellitus |
| Study Design | Randomized controlled trial |
| Country/Setting | Germany/Outpatient diabetes clinics, inpatient diabetes departments |
| Study Duration | NR (7 classes of 45 minutes - timeline unspecified) |
| Inclusion/Exclusion Criteria | Inclusion: insulin-treated diabetes mellitus, at least one geriatric syndrome (such as incontinence), reduced mobility requiring the use of assistive devices, a history of falls during the previous 2 years or cognitive dysfunction), multi-morbidity (more than two chronic diseases besides T2DM), and age >65 years  Exclusion: stroke or myocardial infarction within 2 weeks prior to enrolment, a Mini-Mental State Examination (MMSE) below 18 points implying moderate cognitive dysfunction |
| Sample Size | O: 196; I: 83; C: 72 |
| Intervention Description/Delivery | Description: The SGS comprises seven educational classes of 45 min duration. The SGS DTTP focuses less on theoretical knowledge (pathophysiology, insulin dose adoption, or assessment of carbohydrate intake) but allows a more intensive training of practical capabilities such as insulin injection, self-monitoring and management of hypoglycaemia. The new SGS programme takes into account the changes in learning habits of older people (reduced short-term memory, slowed informational processing). The programme is adapted to the demands of older people, for example by giving instruction more slowly and loudly, avoiding technical terms or giving more intensive practice and using defined numbers of repetitions. Educational materials such as patient books and flipcharts with an adequate type size are provided. In the SGS DTTP, a smaller class size of four to six patients is used, whereas the standard DTTP allows up to 10 people to participate in the education classes.  Delivery: In-person |
| Control Description | The standard DTTP of Berger et al. [5] for insulin therapy takes a period of 5 days with 20 h of training. |
| Follow Up (%) | I: 78; C: 75 |
| Mean Age (SD) | O: 76.2 ± 6.3; I: 75.3 ± 6.2; C: 77.3 ± 6.1 |
| Male (%) | SGS: 30.1; Standard: 37.5 |
| Race (%) | NR |
| SES/Education | NR |
| Type/Duration of Diabetes | Type: T2DM  Duration: Median (range) [years] - O: 12.5 (0-56.5); SGS: 12.5 (0-56.5); Standard: 14.5 (0.3-42.5) |
| Diabetes Treatment | NR |
| Comorbidities | NR |
| Outcome(s) Description | Primary: improvement of metabolic control, self-management skills  Secondary: incidence of acute complications, diabetes knowledge, and treatment satisfaction |
| Type of Analysis | Quantitative |
| Tools for Outcome Assessment | Quality of metabolic control: HPLC Diamat, Munich  Treatment satisfaction and diabetes knowledge: standardized questionnaires  Cognitive function: MMSE, AKT test |
| Frequency of Outcome Assessment | Baseline, immediately after intervention, and 6 months post-DTTP |
| Outcome Results | Knowledge; clinical |
| Study Findings | This study demonstrates that the new SGS DTTP promotes a significant HbA1c decrease, reduces incidence of acute complications, improves diabetes knowledge and enhances diabetes self-management skills. |
| Chadosh Framework Category | Feedback |
